# Managing Local Stressors for Coral Reef Condition and Ecosystem Services Delivery Under Climate Scenarios

**DOI:** 10.3389/fmars.2018.00425

**Published:** 2018-11-09

**Authors:** Mariska Weijerman, Lindsay Veazey, Susan Yee, Kellie Vaché, Jade M. S. Delevaux, Mary K. Donovan, Kim Falinski, Joey Lecky, Kirsten L. L. Oleson

**Affiliations:** 1 Joint Institute of Marine and Atmospheric Research, University of Hawai’i at Mānoa, Honolulu, HI, United States; 2 Pacific Islands Fisheries Science Center, National Oceanic and Atmospheric Administration, Honolulu, HI, United States; 3 Department of Natural Resources and Environmental Management, University of Hawai’i at Mānoa, Honolulu, HI, United States; 4 Gulf Ecology Division, U.S. Environmental Protection Agency, Gulf Breeze, FL, United States; 5 Biological and Ecological Engineering, Oregon State University, Corvallis, OR, United States; 6 Hawai’i Institute of Marine Biology, University of Hawai’i at Mānoa, Kānéohe, HI, United States

**Keywords:** trade-off, ecosystem-based management, multiple stressors, future scenarios, coral reefs, biophysical model, Hawai’i

## Abstract

Coral reefs provide numerous ecosystem goods and services, but are threatened by multiple environmental and anthropogenic stressors. To identify management scenarios that will reverse or mitigate ecosystem degradation, managers can benefit from tools that can quantify projected changes in ecosystem services due to alternative management options. We used a spatially-explicit biophysical ecosystem model to evaluate socio-ecological trade-offs of land-based vs. marine-based management scenarios, and local-scale vs. global-scale stressors and their cumulative impacts. To increase the relevance of understanding ecological change for the public and decision-makers, we used four ecological production functions to translate the model outputs into the ecosystem services: “State of the Reef,” “Trophic Integrity,” “Fisheries Production,” and “Fisheries Landings.” For a case study of Maui Nui, Hawai’i, land-based management attenuated coral cover decline whereas fisheries management promoted higher total fish biomass. Placement of no-take marine protected areas (MPAs) across 30% of coral reef areas led to a reversal of the historical decline in predatory fish biomass, although this outcome depended on the spatial arrangement of MPAs. Coral cover declined less severely under strict sediment mitigation scenarios. However, the benefits of these local management scenarios were largely lost when accounting for climate-related impacts. Climate-related stressors indirectly increased herbivore biomass due to the shift from corals to algae and, hence, greater food availability. The two ecosystem services related to fish biomass increased under climate-related stressors but “Trophic Integrity” of the reef declined, indicating a less resilient reef. “State of the Reef” improved most and “Trophic Integrity” declined least under an optimistic global warming scenario and strict local management. This work provides insight into the relative influence of land-based vs. marine-based management and local vs. global stressors as drivers of changes in ecosystem dynamics while quantifying the tradeoffs between conservation- and extraction-oriented ecosystem services.

## INTRODUCTION

Coral reef ecosystems provide valuable resources. They buﬀer coastal erosion, provide a cornucopia of food resources, attract tourism dollars, supply construction and pharmaceutical materials, and provide recreational opportunities for humans and essential habitat for threatened and endemic organisms (Hoegh-Guldberg, 1999; Moberg and Folke, 1999; Spalding et al., 2017). Furthermore, nature-based solutions, such as using living reefs as natural barriers for storm protection, are more cost-eﬀective than manufactured infrastructure (Daily and Matson, 2008).

Despite the importance of reef ecosystems, they are under threat on a local scale from coastal development, overfishing, invasive species, and pollution, and on a global scale from ocean acidification, warming, and hypoxia (Carlton and Scanlon, 1985; Jokiel and Coles, 1990; Pörtner et al., 2005; Hoegh-Guldberg et al., 2011; Prouty et al., 2014). Two extensive reviews on threats to coral reefs identified ocean warming and ocean acidification as prominent threats (Burke et al., 2011; Brainard et al., 2013). Increasing carbon dioxide (CO_2_) emissions are slowly causing the world’s oceans to become warmer and more acidic. Ocean acidification reduces calcification rates of all calcifying organisms including corals. Intense or prolonged ocean warming can result in the expulsion of the symbiotic algae that live in the coral tissue leaving them looking “bleached” and is hence called coral bleaching. Bleached corals have a higher change of mortality as they become more susceptible to pathogens (Maynard et al., 2015b). These threats are projected to intensify in coming decades (Van Hooidonk et al., 2013; Maynard et al., 2015b). Chronic stressors can lead to a more degraded reef system that has tipped to an algal dominated benthos (Bellwood et al., 2004; Hughes et al., 2010), and a replacement of top predatory fishes (large slow growing fishes) with species with a high turnover (Heithaus et al., 2008; Ruttenberg et al., 2011; Maynard et al., 2015a). These shifts are a concern because ecological functions and economic values diminish on such reef systems. Eﬀective, long-term conservation of coral reefs and the goods and services they provide requires addressing the most critical threats.

The development of policies to address threats and promote ecosystem services is dependent on an understanding of ecosystem dynamics and responses to major stressors. Ecosystem models can synthesize the present-day condition and project changes of a system as a result of management regulations, climate conditions, or human use. Spatially explicit ecosystem models can also quantify the relative impacts of land-based vs. marine-based threats (e.g., land-based pollutants vs. fishing; (Álvarez-Romero et al., 2011; Barbier et al., 2011) and local vs. global stressors (Gurney et al., 2013; Weijerman et al., 2015). These types of models can evaluate tradeoﬀs of alternative courses of action to mitigate threats (Hulme, 2005; Fung, 2009; Weijerman et al., 2016). More recently, ecosystem models have been coupled with economic concepts to translate ecological outcomes in terms of human wellbeing, such as ecosystem services (Orlando and Yee, 2017).

One such spatially-explicit, biophysical ecosystem model is the COral Reef Scenario Evaluation Tool, CORSET (Fung, 2009; Melbourne-Thomas et al., 2011a; Principe et al., 2012). It includes hydrodynamics (which defines the connectivity), ecological dynamics, and land-based (nutrient and sediment pollution) and marine-based (fishing) stressors as well as global climate-related stressors (hurricanes and ocean warming). Its main use is to evaluate tradeoﬀs of alternative management or climate-related scenarios (Melbourne-Thomas et al., 2011b). Building on the extensive work to estimate nutrient and sediment loads and fish extraction on a 500 × 500 m scale around the main Hawaiian Islands (Wedding et al., 2017), we were able to incorporate these local stressors into the adapted CORSET model, the Hawai’i Reef dynamics Simulator or HIReefSim. Additionally, annual bleaching events were projected to start between 2035 and 2045 for the main Hawaiian Islands (Van Hooidonk et al., 2016). These projections were based on the results of an ensemble model of Intergovernmental Panel on Climate Change, Coupled Model Intercomparison Project Phase 5 (CMIP5), and as such, incorporated spatial variability in the eﬀects of ocean warming on coral reefs. We used the projection of the Representative Concentration Pathway (RCP) 8.5 which estimates that by 2040 CO_2_ emissions have reached 480 ppm and the onset of annual bleaching has begun (Van Hooidonk et al., 2016).

While ecological indicators are being used explicitly in management and policy (Arkema et al., 2006; Levin et al., 2013), decision-makers and the public often relate more to direct experiences, such as fishing, recreation, or coastal protection (Yee et al., 2014). Goods and services provided by coral reef ecosystems have long been acknowledged (e.g., Moberg and Folke, 1999), however, the relatively recent field of ecosystem service modeling quantifies these direct benefits to humans from functioning ecosystems (Bagstad et al., 2013). One approach uses “ecological production functions” (EPFs) to translate environmental shifts into economic implications in a way that is meaningful to decision-makers and resource managers (Nelson et al., 2009; Orlando and Yee, 2017). EPFs calculate the provision of goods and services as a function of specific ecological attributes (de Groot et al., 2002). Defining an EPF relies on an ecological understanding of which attributes are important to ecological function, as well as an economic understanding of what functions are valuable to humans. While an EPF quantifies the potential supply of ecosystem goods and services based on ecosystem condition, the realized value will depend on human demand and access (Wainger and Boyd, 2009). Economic valuation requires another relationship, ecosystem service valuation functions, to derive the value society gets from direct (e.g., food and recreation) and indirect (e.g., shoreline protection) use and non-use (e.g., existence) of these goods and services (Compton et al., 2011; Yee et al., 2014). In this way, EPFs can be used to evaluate changes in potential provision of goods and services due to management, climate, and human use that aﬀect the ecosystem, while valuation functions can calculate the cost/benefit of those changes.

To evaluate how diﬀerent local management approaches [sediment mitigation and marine protected areas (MPA) establishment] could improve the provision of coral reef ecosystem goods and services, an ecosystem model that simulates impacts of both land- and marine-based management was parameterized for Maui Nui, Hawai’i, i.e., the islands of Maui, Lāna’i, Moloka’i, and Kaho’olawe. These management approaches were also combined with two future severities of climate-related stressors. Reefs of Maui Nui served as a case study, but this tool can be used in other areas with similar local and global threats (Melbourne-Thomas et al., 2011a; Kapur and Franklin, 2017). Although several studies have shown the mitigating eﬀects of local management on coral degradation in the face of climate change (Hughes et al., 2007; Kennedy et al., 2013; McClanahan et al., 2014), other studies have shown that under a “business as usual” greenhouse gas emissions future (IPCC RCP8.5 trajectory), local management may be unable to prevent further degradation of coral reef ecosystems (Thompson and Dolman, 2010; Selig et al., 2012; Weijerman et al., 2015; Hughes et al., 2017).

Here, we ask two questions: (1) What is the relative importance of land- and marine-based management action? and (2) Can local management mitigate the eﬀects of climate-related stressors? We expect that a combination of proactive local actions (sediment mitigation and fisheries controls) will attenuate declines in coral reef ecosystem goods and services delivery, but without local management, reefs will continue to decline, a trend exacerbated with more extreme future climate conditions.

## METHODS

### Study Region

**FIGURE 1 |:**
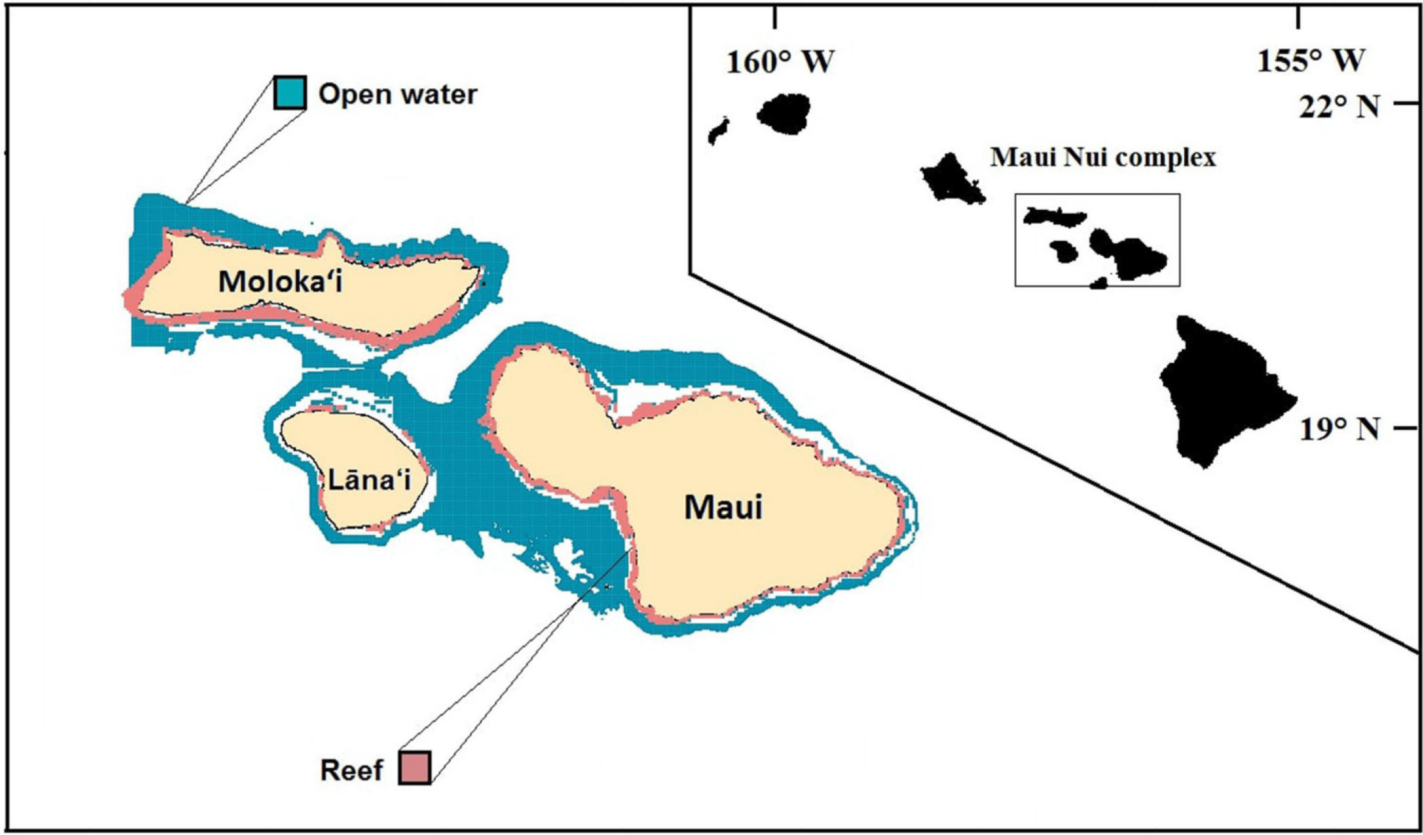
Modeled area of the Maui Nui complex consisting of the Hawaiian Islands: Maui, Moloka’i, and Lāna’i, with the inset figure showing the location of Maui Nui in the Hawaiian Archipelago. The pink “reef” area is the 0–30 m depth range included in the model. The outer edge of open water area (blue) is defined by the 200 m depth contour. White areas interior of this indicate gaps in bathymetry data.

Our study area encompasses ∼325 km^2^ of shallow coral reef habitat across the Hawaiian Islands of Maui, Molokai and Lāna’i, i.e., Maui Nui (Figure 1; the island of Kaho’olawe is excluded from this analysis due to lack of data). Of this area, 12 km^2^ (3.6% of total reef area) are classified as MPAs, with just over 9 km^2^ of the protected areas being designated as “no-take” area. The model domain consists of the shallow (0–30 m) reef zone around Maui Nui and is spatially represented by a 500 × 500 m grid cell network.

### HIReefSim

HIReefSim (Hawai’i Reef dynamics Simulator) is based on the framework of the Coral Reef Scenario Evaluation Tool (CORSET) developed by Fung (Fung, 2009) and adapted by Melbourne-Thomas et al. (2011a) and Principe et al. (2012). Model components include (1) 500 × 500 m gridded basemaps of the study region (see details below); (2) model dynamics (see details below); (3) larval connectivity zone delineations, which detail transition probabilities between larval sources and sinks; and (4) hurricane zone delineations, which were designed to represent grouped swaths of coastline that are similarly aﬀected by storm events. Modeled stressors include land-based sediment and nutrient input, fishing, hurricane damage to corals and macroalgae, and climate-related coral mortalities due to coral bleaching.

### Basemaps

The HIReefSim model defines two consumer functional species groups, herbivorous (algal grazers) and piscivorous (predatory) fishes, and five benthic functional groups: macroalgae, turf algae, crustose-coralline algae (CCA), and spawner and brooder corals. Boosted regression trees generated spatial predictive maps of these ecological variables based on observations from a compilation of underwater surveys and an extensive gridded predictor dataset (Table S1) (Stamoulis et al., 2016; Delevaux, 2017). Unlike in the instantiation of CORSET, urchins and large (>60 cm) piscivores were not included due to very low abundance of large piscivores and a lack of urchin data preventing the creation of predictive maps.

### Dynamics

**FIGURE 2 |:**
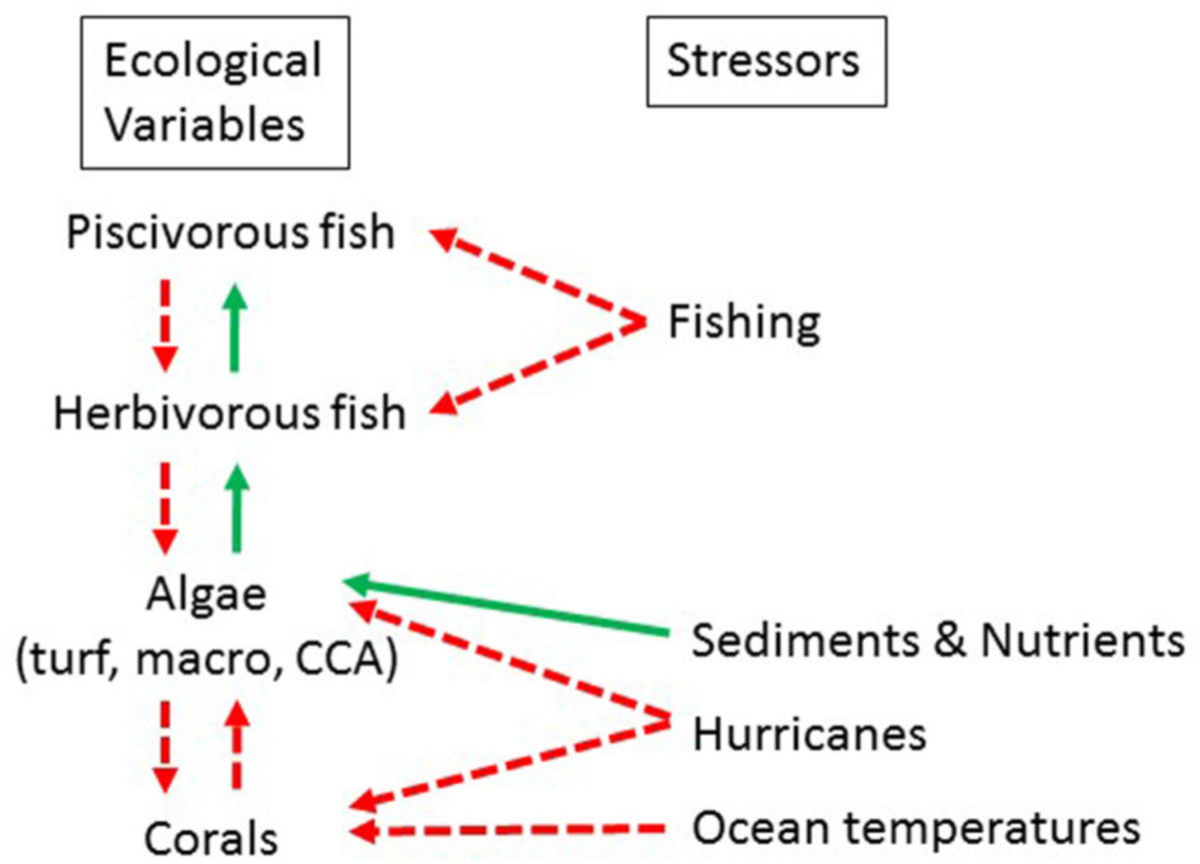
Dynamic relationships between ecological variables and external stressors to the variables. A solid green arrow indicates a positive relationship and a dashed red arrow indicates a negative one.

Coral reef ecosystems are extremely complex systems and influenced by a myriad of variables. HIReefSim only includes key ecological dynamics by using diﬀerential equations to estimate the interactions among the functional groups and their response to stressors in each grid cell (Supplementary Text S2). For example, ocean warming has led to global degradation of coral reefs with a consequent loss of structure followed by a decrease in fish biomass (Alvarez-Filip et al., 2009; Graham and Nash, 2013). Additionally, on a local scale, an increase in nutrients leads to an increase in the faster-growing macroalgae which in turn can reduce the growth of corals and impede coral recruitment. These are the key dynamics that are incorporated in the model (Figure 2), other stressors to coral reef ecosystems (e.g., ocean acidification, hypoxia, invasive species) are not included. Fung (2009) and Melbourne-Thomas et al. (2011a) give detailed descriptions of the model development, general model behavior, and sensitivity analyses. Kapur and Franklin (2017) describe the applicability of the CORSET model for Hawaiian reef systems. Here, only the main components of CORSET that form the basis for HIReefSim input are described (Supplementary Text S2 has details of model equations and parameter estimates). Estimates of ecological variables represent the current (∼2004–2012) reef condition. The model has some stochasticity as it randomly selects the intensity, frequency, and region of impact to capture year-to-year variation in storms. The larval connectivity matrices were based on a pelagic duration of 45 days, calculated at the 50 m depth layer, for both corals and fishes (Wren and Kobayashi, 2016).

### Modeled Stressors

Spatially explicit stressor functions included nutrification and sedimentation (changes modeled via parameter scaling), fishing (modeled as a spatial explicit reduction in fish biomass), and coral and macroalgal mortality resulting from wave action (severity dependent on hurricane zone; Figure 2; Table 1). The projected increase in bleaching-related coral mortalities was a non-spatial stressor, aﬀecting all corals equally.

### Model Adaptations

A regional study using CORSET (Kapur and Franklin, 2017), concluded a seemingly sustainable herbivore fishery would be possible despite the projected decline in coral cover. However, a limitation of this simplified model is its broad groupings of fishes in just herbivores and piscivores and the lack of two-way dynamics in fish size and fishing eﬀort. For example, model results show that herbivores increase but the composition of this group is likely dominated by large-bodied herbivores, such as the larger surgeonfishes (e.g., *Acanthurus dussiemeri, A. xanthopterus*), that escape piscivore predation because of their size. However, if fishing is not restricted, these larger-bodied fishes are key targets for spearfishers and an increase in spearfishing is not accounted for in the model. Additionally, reef structure is likely to erode due to coral cover decline, preventing fish recruits and juveniles from hiding (Alvarez-Filip et al., 2009; DeMartini et al., 2010; Graham and Nash, 2013) and increasing their accessibility to their predatory fishes (Rogers et al., 2014), ultimately leading to a decline in reef fish productivity (Gratwicke et al., 2005). Therefore, we included two scalars related to the structural complexity a coral reef provides for: (1) the survival of both herbivorous and piscivorous fish recruits given by the relationship *survival (Frec*) = *aC* / [1 + (*a/b)* ^∗^ *C*^*d*^], where *C* is coral cover, and *a, b* and *d* are fitted parameters (Table S2 in Supplementary Text S2; Gurney et al., 2013) and (2) the susceptibility of small and juvenile herbivorous fishes to predation with high coral cover leading to more hiding spaces and hence lower susceptibility to predation with the relationship *refuge*= *min*(*H,F*_*pred*_^∗^*C*)], where *C* is coral cover and *H* herbivore biomass and *F*_*pred*_ a fitted parameter (Table S2 in Supplementary Text S2) (Liu and Xing, 2012; Rogers et al., 2014).

### Model Calibration and Validation

The model was validated using a two-fold approach (Melbourne-Thomas et al., 2011a,b):

- Calibrate model parameters to reproduce a community structure typical of stable, “healthy” Hawaiian reef in the absence of external stressors over long-term trajectories (40 years); and
- Evaluate whether the model can reproduce historic broad-scale dynamics for the Maui Nui region over the past 30 years (1985–2015), given a timeline of known stressors (Supplementary Text S3). Historic land-based stressors (derived from land use maps in 1920) were scaled to present-day values (Supplementary Text S3).

Results of model validation are presented in Supplementary Text S3. During calibration, it became apparent that the model was very sensitive to the parameters related to fish growth. For herbivores this was grazing pressure (*gt*, *gm*) and the biomass accumulation from grazing (*mm*, *mt*, *me*), and for piscivores this was prey availability (*iph*), predation pressure (*gp*) and biomass accumulation from predation (*rp*). We therefore randomly selected 50 values between the estimated ranges (Table S2) and assumed a normal distribution and ran each scenario 50 times to obtain uncertainty estimates related to these parameters.

### Scenario Simulations

Fifteen scenarios of separate and coupled eﬀects of climate-related stressors and management actions were simulated (Table 2). To define values for future baseline stressors (sediment and nutrient influx, herbivore and piscivore catches), annual projected population growth of 0.8% was used (DBEDT, 2016). Fishing pressure, along with sediment and nutrient runoﬀ, was assumed to increase proportionally with the projected population growth. These stressors were further adjusted as specified within the scenario (Table 2).

To simulate climate change, we focused on hurricanes and bleaching-related coral mortality events. Both the frequency and intensity of cyclones in the North Pacific have increased, and sea surface temperatures (SST), which are directly correlated with storm intensity, are increasing as well (Emanuel, 2005). Murakami et al. (2013) project an average 267% increase in the number of cyclones that will reach the main Hawaiian Islands between 2075 and 2099 (0.75 annually increasing to 2 annually). To reflect these projections, we included hurricane events which directly impact Maui Nui an average of every 10 years for the running period of the climate change scenarios (Table 2). We specified that each hurricane event would reduce coral and macroalgae cover by 49% based on the average observed reduction in living bottom cover across the west coast of Hawai’i island (Dollar and Tribble, 1993). With the projected increase in sea surface temperatures, bleaching events will likely become annual, seasonal occurrences in the next 15–25 years (Van Hooidonk et al., 2016). Taking into consideration the variability in these estimates (Peters et al., 2017), we modeled a “severe” climate-related stressor scenario where we assumed that annual bleaching is every other year and a “less severe” climate-related stressor scenario where we assumed two annual bleaching events per decade.

### Ecological Production Functions (EPFs)

We applied four Ecological Production Functions (EPFs): “State of the Reef ” (unitless), “Trophic Integrity of the Reef ” (unitless), “Fisheries Production” (i.e., resource fish biomass in kg/km^2^), and “Fisheries Landings” (i.e., annual fish catch in kg/km^2^) to translate HIReefSim model output into values important for management applications (Principe et al., 2012; Yee et al., 2014). These EPFs represent a supporting service (first two EPFs), a potential provisioning service, and an actual provisioning service (Millennium Ecosystem Assessment, 2005), respectively, and roughly relate to biodiversity, ecosystem structure and function, conservation, and food yield outcomes. For each of the EPFs, the relative change from end to start of the simulation period (40 years) under the two climate change scenarios was calculated. To assess eﬀectiveness, the relative change between the alternative management scenarios and current management was calculated. Based on model validation where the mean value of the 50 simulations described historical coral cover trajectory well (Supplementary Text S3), we used the scenario means for each parameter that described the EPF (see below) for each reef cell. We then present the resulting EPF values as a mean (and standard error) for all reef cells. We also show the spatial variation of each EPF under the diﬀerent scenarios visually in maps.

### State of the Reef

The ecological status of Maui Nui reefs was represented by the “State of the Reef,” a supporting ecosystem service defined as:

(1) $$\sum_{i=1}^{5}w_{i}\times R_{i}$$

where *w*_*i*_ is the weighting factor of each *R*_*i*_, with *R*_*i*_, representing the standardized value of five key indicators of reef structure: coral cover, macroalgal cover, total fish biomass, fish richness, and coral richness (Supplementary Text S4). An expert survey defined weighting factors as: (i) coral cover 30%; (ii) coral richness 20%, (iii) fish biomass 20%; (iv) fish richness 15% and (v) macroalgal cover 15% (Van Beukering and Cesar, 2004). Coral richness and fish richness values were based on Orlando and Yee (2017). The ecological indicator scores were scaled to the maximum value of each indicator.

### Trophic Integrity

The trophic integrity was estimated by the ratio of calcifiers [corals (*C*) and CCA] and fleshy algae [turf (*T*) and macroalgae (*MA*)] and the trophic level of the fish community with herbivores (*H*) having a trophic level of 2 and piscivores (*P*) a trophic level of 4:

(2) $$0.5*\left(\frac{C+CCA}{T+MA}\right)+0.5*\left(2*\frac{H}{H+P}+4*\frac{P}{H+P}\right)$$

Ecological indicator scores were scaled to the maximum value of each indicator.

### Fisheries Production

Present-day predicted biomass of resource fish species (defined as species that had ≥ 450 kg of average annual harvest from 2000 to 2010 in the state of Hawai’i) was calculated as a ratio of total fish biomass (McCoy et al., 2018). This ratio was assumed to remain constant in the course of fluctuations in total fish biomass. Fisheries Production, therefore, represents the biomass of resource fish, a potential ecosystem service.

### Fisheries Landings

The model directly calculates fish catch as a function of available fish biomass (basemap input layer) maximum fishing eﬀort, and accessibility (last two dynamics estimated for Maui Nui, Supplementary Text S3). Fisheries Landings is a provisional ecosystem service.

## RESULTS

### Local Management Strategies Can Attenuate Declines in Ecological Outcomes

**FIGURE 3 |:**
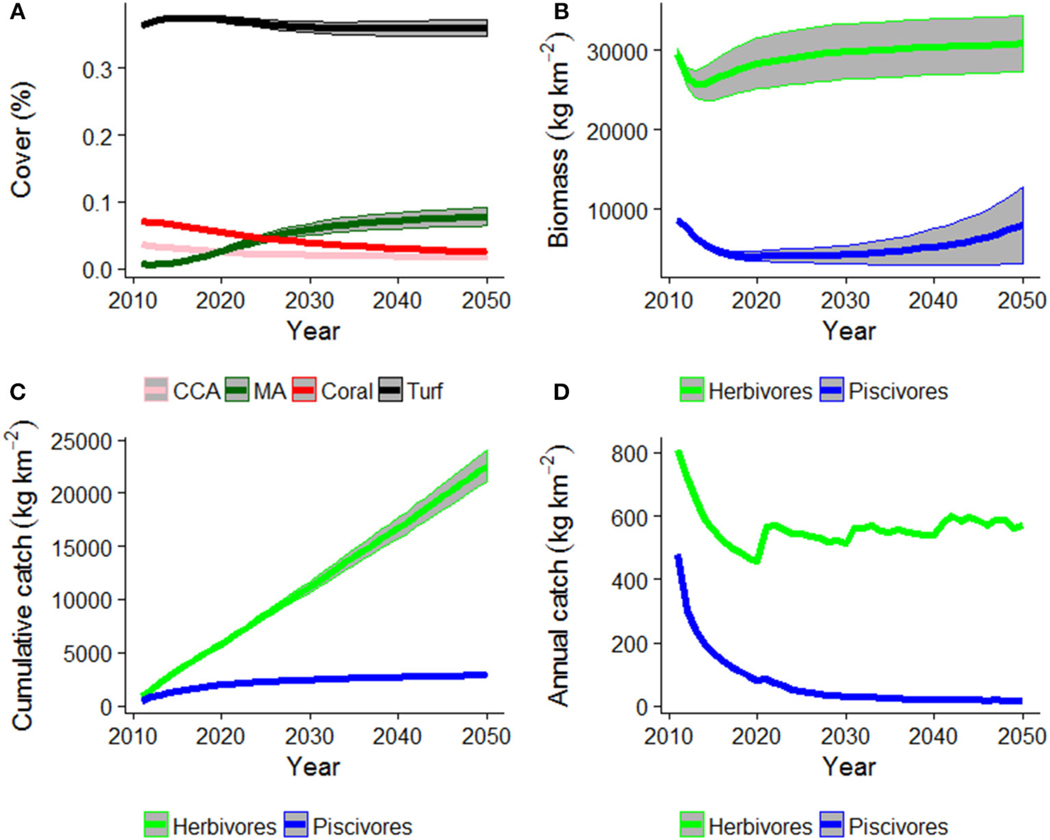
HIReefSim model estimated trajectories of **(A)** cover of crustose-coralline algae (CCA), macroalgae (MA), coral, and turf algae, **(B)** fish biomass, **(C)** fish cumulative catch, and **(D)** annual catches under the Current Management scenario. Shaded area is ± 1 standard error of the mean.

Under the Current Management scenario, coral cover declined and was replaced by macroalgal cover (Figure 3A). Piscivore biomass also declined, to less than half the initial biomass but could recover in the last decade (Figure 3B), likely due to a shift in catch composition to predominantly herbivorous fishes (Figures 3C,D).

**FIGURE 4 |:**
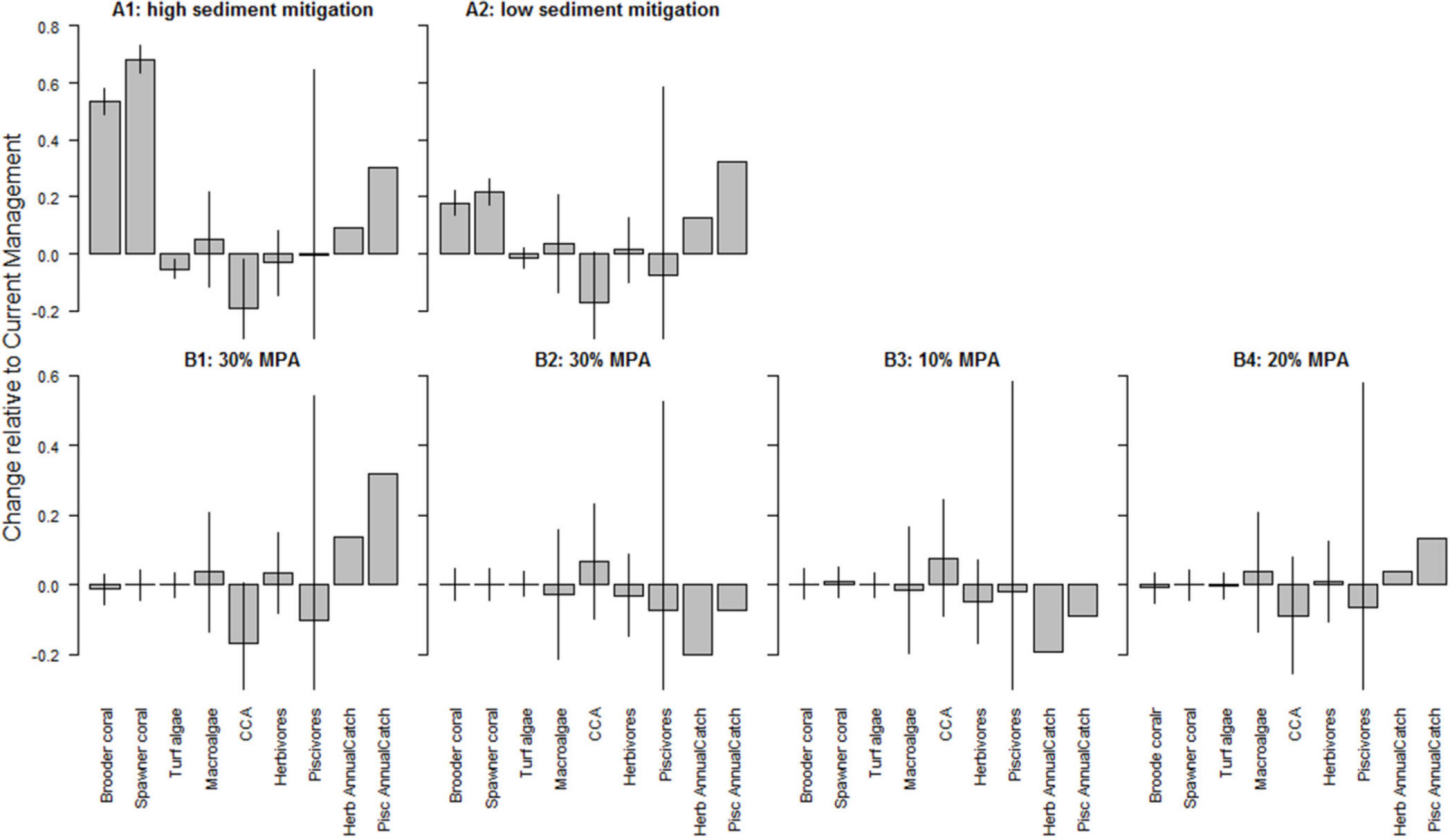
Changes in benthic cover (corals and algae) and fish (herbivores and piscivores) biomass compared to Current Management as a result of local management including (*top row*) high (A1) and low (A2) sediment mitigation and (*bottom row*) additional MPA establishments with B1 and B2 additional 30% randomly selected MPAs, B3 10% additional MPAs and B4 20% additional MPAs. A value of 0 indicates no change from the outcome in 2050 under Current Management, values > 0 indicate an improvement (or, more accurately, less of a decline from the Current Management), while values < 0 indicate a worsening of the decline relative to Current Management. The bars represent ± 1 standard error as a percentage of the mean. Annual catch was calculated post model simulations based on the results of cumulative catches of the 50 simulations per scenario and hence has no error bar. CCA is crustose coralline algae.

A continuation of current management attenuated the downward trend in coral cover. Comparing land-based management scenarios (high and low sediment mitigation, A scenarios) and marine-based management scenarios (additional no-take MPAs, B scenarios), sediment mitigation strongly impacted benthic composition, whereas fisheries management impacted fish biomass (Figure 4). For example, reduction of sediment input slowed the decline in coral cover compared to Current Management (Supplementary Figures S5.1, 5.2). Sediment mitigation exhibited a mixed eﬀect on algal cover: it limited the space occupied by turf algae and CCA, but it had negligible eﬀect on macroalgal cover (Figures S5.3–5.5). Fish biomass also responded to a change in benthic composition. In the high mitigation sediment reduction scenario (A1), herbivorous fish biomass declined slightly compared to Current Management (Figure S5.6) whereas piscivore biomass declined slightly under the low mitigation scenario (A2).

Marine-based management strategies had minimal impact on the trajectories of coral cover compared to the Current Management scenario (Figure 4, Figures S5.1, 5.2). CCA, however, did respond to MPA designations with decreases under one of the 30% MPA scenario (B1) and 20% MPA scenario (B4) but increased slightly under the other 30 and 10% MPA scenario (B2, B3 resp.; Figure 4, Figure S5.5). Interestingly, fish biomass hardly changed under MPA scenarios (Figure 4, Figure S5.6). The large error bar on the piscivore biomass also reflects the sensitivity of the model to the parameterization of fish-related variables.

The expansion of current MPA boundaries to include areas encompassing the top 10% (B3) and 20% (B4) of fish biomass and coral cover or the randomly placed 30% MPAs (B1 and B2) showed no clear pattern in the results. Striking is that the placement of the 30% no-take MPAs had opposite outcomes for fish catch which declined by up to 20% under B2 and increased to 32% under B1 (Figure 4) for herbivore and piscivore catches, respectively.

### Local Management Strategies Have Mixed Results Under Climate-Related Stressor Scenarios

**FIGURE 5 |:**
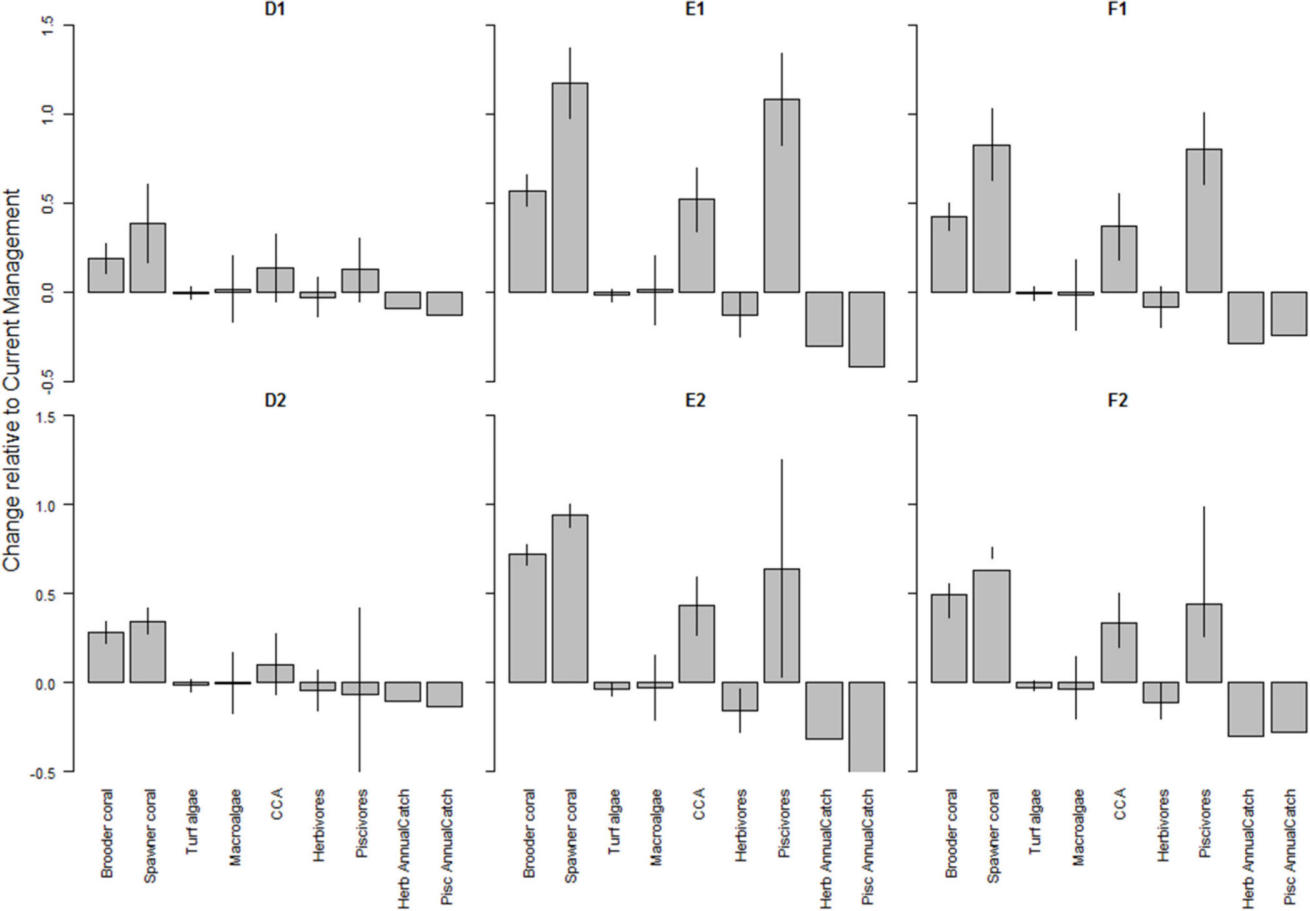
Changes in benthic cover (corals and algae) and fish (herbivores and piscivores) biomass as a result of local management under (*top row*) severe climate-related stressors and (*bottom row*) less severe climate-related stressors. A value of 0 indicates no change from the outcome in 2050 under Current Management, values > 0 indicate an improvement (or, more accurately, less of a decline from the Current Management), while values < 0 indicate a worsening of the decline relative to Current Management. The bars represent ± 1 standard error as a percentage of the mean. The local management scenarios include: (D) 10% no-take MPAs and low sediment mitigation, (E) 30% MPAs and strict sediment mitigation, and (F) 30% MPAs with an additional 20% reduced piscivore fishing effort and strict sediment mitigation. CCA is crustose coralline algae.

Model projections resulted in steep declines in coral cover from current levels, especially under the more severe climate-related stressors scenarios where hurricanes and thermal stress led to coral mortality (Figures S5.1, 5.2). Local management did somewhat mitigate the eﬀects of a changing climate, with slightly more benefit (i.e., lower net loss) under the more severe scenario (Figure 5). Climate-related stressors were projected to have a positive eﬀect on herbivorous fish biomass (Figure S5.6 “base”), while local management buﬀered losses in piscivorous fish biomass, which then resulted in decreased herbivore biomass especially under the stricter management scenarios (E and F; Figure S5.6 “scenario”). However, due to the large fluctuations in fish biomass, these diﬀerences in herbivore biomass were not statistically significant (Figure 5). Under all climate-related stressors and management scenarios (D-F), herbivores increased with 5–11 t/km^2^ across Maui Nui from 2015 to 2050 (Figure S5.6). These increases represent an ecologically valid outcome, given the overall increases in turf and macroalgal cover, which are food sources for herbivorous fishes (Figure 2). Hurricanes did result in temporary “dips” in the overall increase in macroalgal cover under both climate-related stressors scenarios but due to their relatively high growth rate, macroalgae recovered within a year. In general, under the climate scenarios, local management benefited corals and piscivores with a tradeoﬀ in fisheries catches. These results were more pronounced under the stricter management scenario E and F, and under the higher climate stress scenario (Figure 5).

### Implications for Ecosystem Goods and Services

**FIGURE 6 |:**
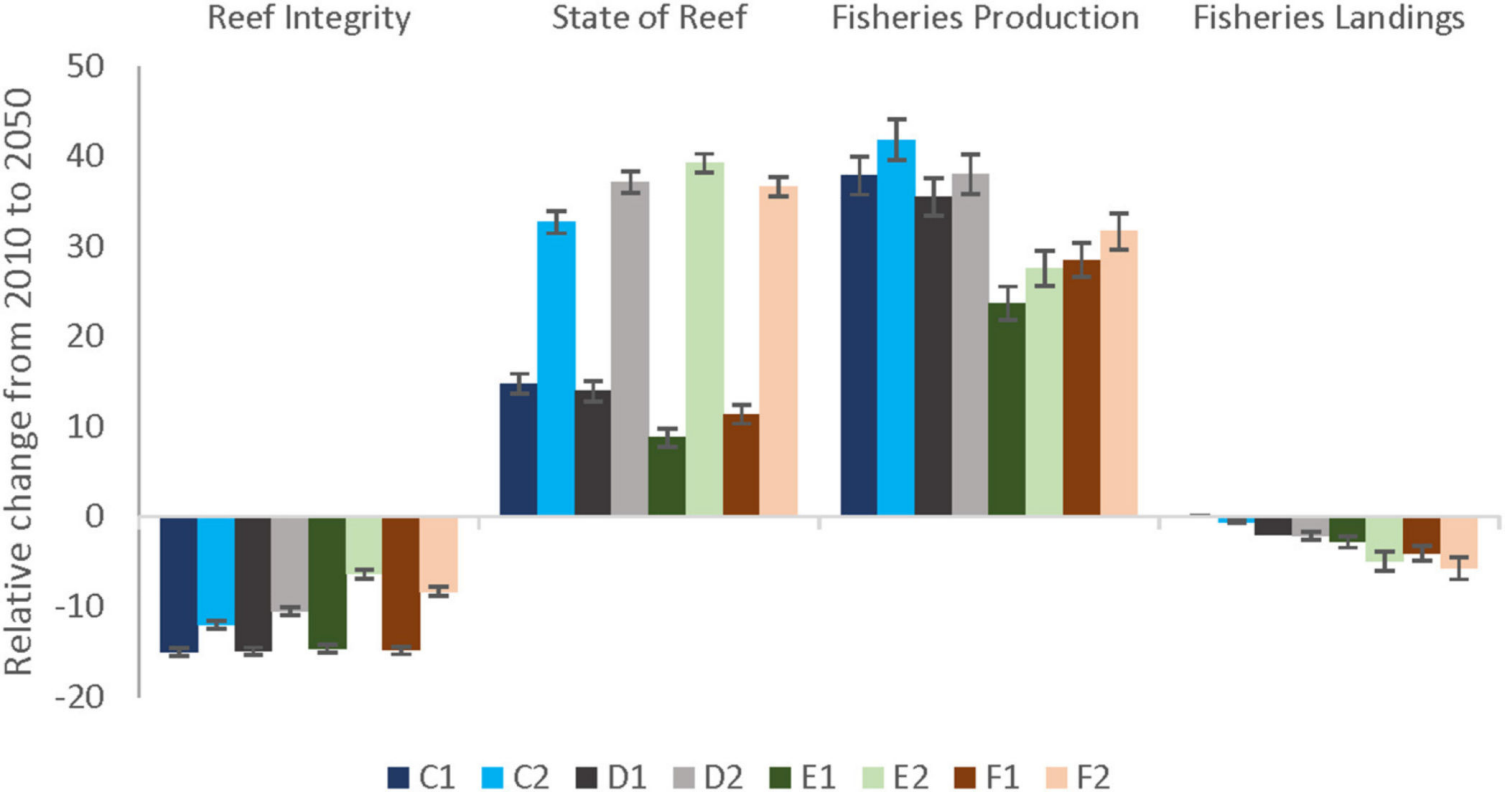
Relative change (%) from 2010 to 2050 of each scenario including severe (darker bars) and less severe (lighter bars) climate change impacts. The blue bars (C1, C2) represent a future projection of Current Management practices, population growth, and severe (bleaching-related coral mortalities every other year; C1) and less severe (mortalities every 5 years; C2) climate-related stressors, respectively. The effects of local management are also shown under two climate scenarios: severe climate-related stressors in darker colors (denoted by a 1) and less severe stressors in lighter colors (denoted by a 2). The local management scenarios include: (D–gray bars) 10% no-take MPAs and low sediment mitigation, (E– green bars) 30% MPAs and strict sediment mitigation, and (F–red bars) 30% MPAs with an additional 20% reduced piscivore fishing effort and strict sediment mitigation.

Under a future scenario of severe climate stress and current management (C1, Figure 6), Trophic Integrity declined by 15% and Fisheries Landings by 6%. However, in the entire Maui Nui area, State of the Reef and Fisheries Production increased by up to 41%, but with large spatial variation. Across all scenarios, local management improved the Trophic Integrity of the reef (or dampened the decline) and the State of the Reef under the less severe climate change scenarios but had slightly negative eﬀect on State of the Reef under severe climate change as well as on Fisheries Production and Fisheries Landings (Figure 6).

Local management could not prevent a decline in the Trophic Integrity; however, it did decrease the trajectory. Comparing Current Management (C1) to increasingly stringent local management of sediment and fishing stressors under severe climate-related stressors (D1, E1, and F1; darker colors in Figure 6), shows a trend of diminished decline in Trophic Integrity. Even larger improvements were evident under less severe climate-related stressors.

State of the Reef and Fisheries Production displayed improved results under all scenarios due to the increase in herbivore biomass that are components of these EPF. Model results showed a counterintuitive trend with management as both EPFs had lower values under severe climate scenarios compared to current management and Fisheries Production also decreased under less severe climate change. These lower values can be attributed to the smaller decreases in piscivore biomass due to management, leading to more predation pressure on herbivorous fish (Supplementary Figures S5.6, S5.7). On the other hand, State of the Reef clearly improved with local management under the less severe climate change scenarios (Figure 6).

Fisheries Landings were projected to decrease more compared to current management (C), especially under the scenarios including 30% MPAs (E, F). Due to the low piscivore biomass, an additional 20% reduced piscivore pressure (F) had almost no additional eﬀect in landings (Figure 6).

**FIGURE 7 |:**
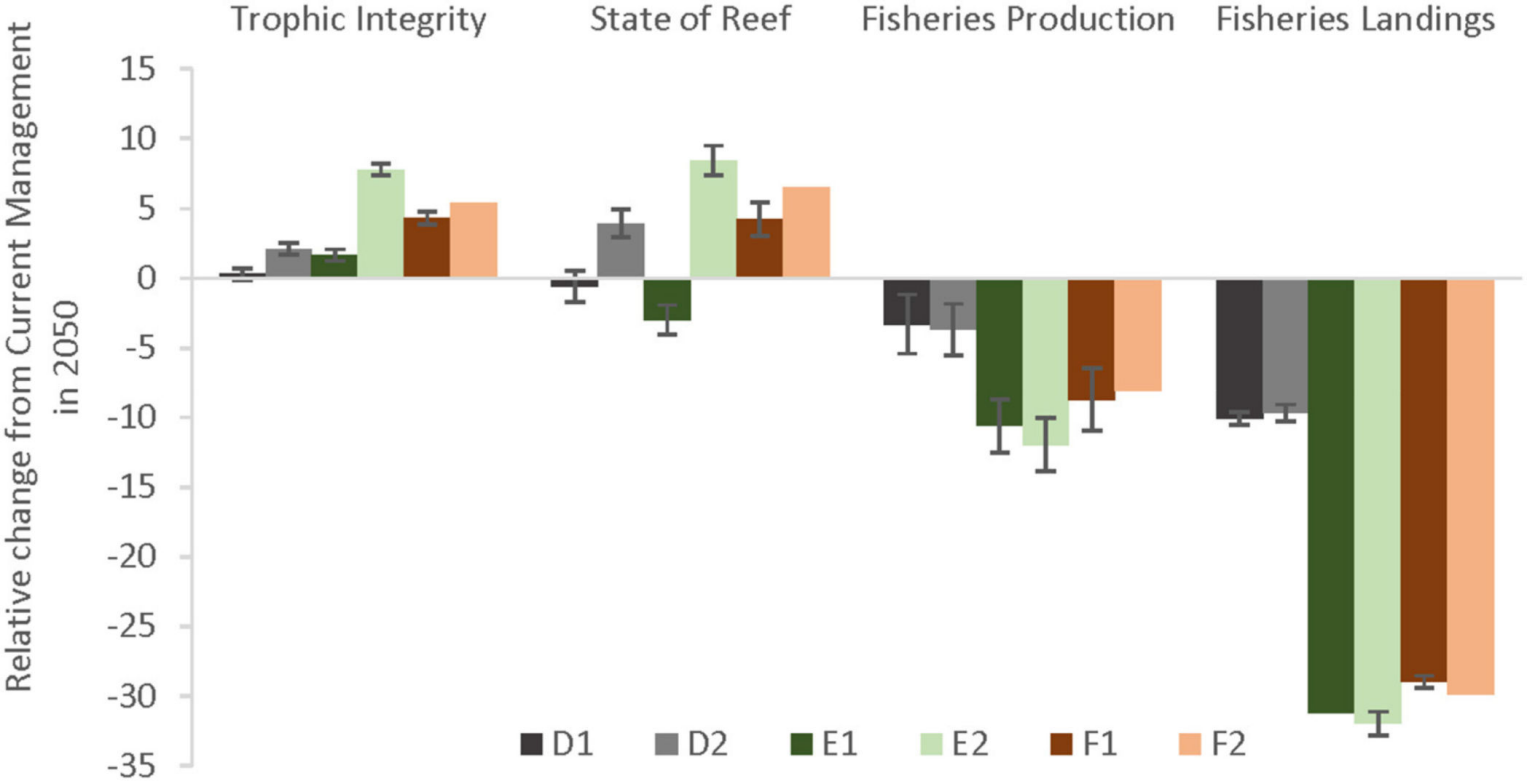
Effectiveness (% change) of additional management compared to Current Management in 2050 under two climate change scenarios. Severe climate-related stressors are represented by darker colors (denoted by a 1 in the scenario letter abbreviations) and less severe stressors in lighter colors (denoted by a 2). The local management scenarios include: (D–gray bars) 10% no-take MPAs and low sediment mitigation, (E–green bars) 30% MPAs and strict sediment mitigation, and (F–red bars) 30% MPAs with an additional 20% reduced piscivore fishing effort and strict sediment mitigation.

Looking at the eﬀectiveness of local management scenarios by comparing the end states of the EPFs of each scenario relative to current management, both the Trophic Integrity of the reef as well as the State of the Reef can benefit from additional management especially under less severe climate change (Figure 7). Fisheries Production fared less well, likely because of the increase in piscivore biomass (resulting in less herbivores) and the largest tradeoﬀ was in Fisheries Landings.

**FIGURE 8 |:**
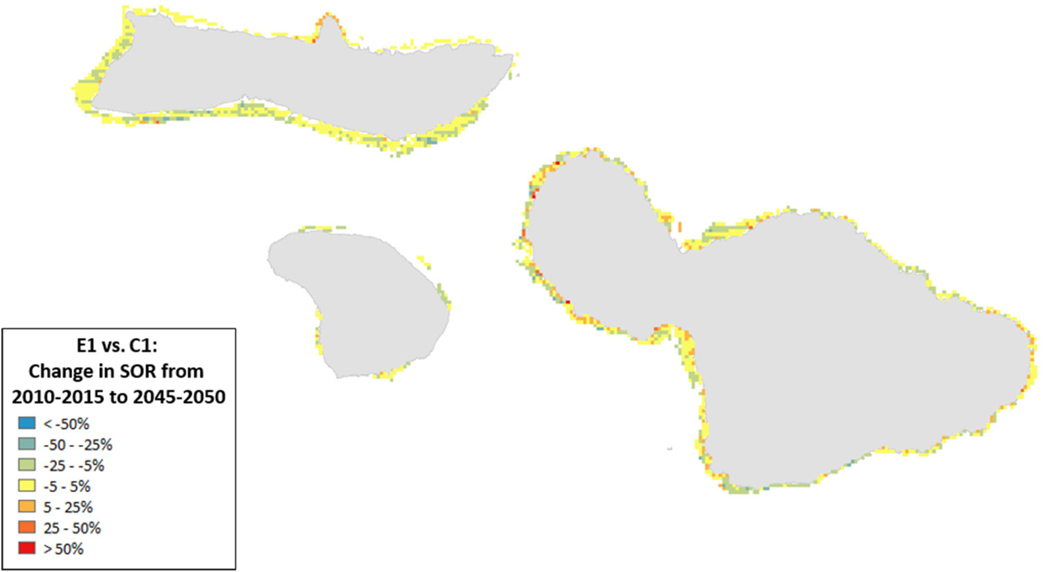
Ratio of the change in State of the Reef (2010–2050) for scenario E1 (high sediment mitigation, 30% MPAs) compared to C1 (Current Management) under severe climate-related stressors. A value of 0 indicates no change from the outcome in 2050 under Current Management, values > 0 indicate an improvement (or, more accurately, less of a decline from the Current Management), while values < 0 indicate a worsening of the decline relative to Current Management.

**FIGURE 9 |:**
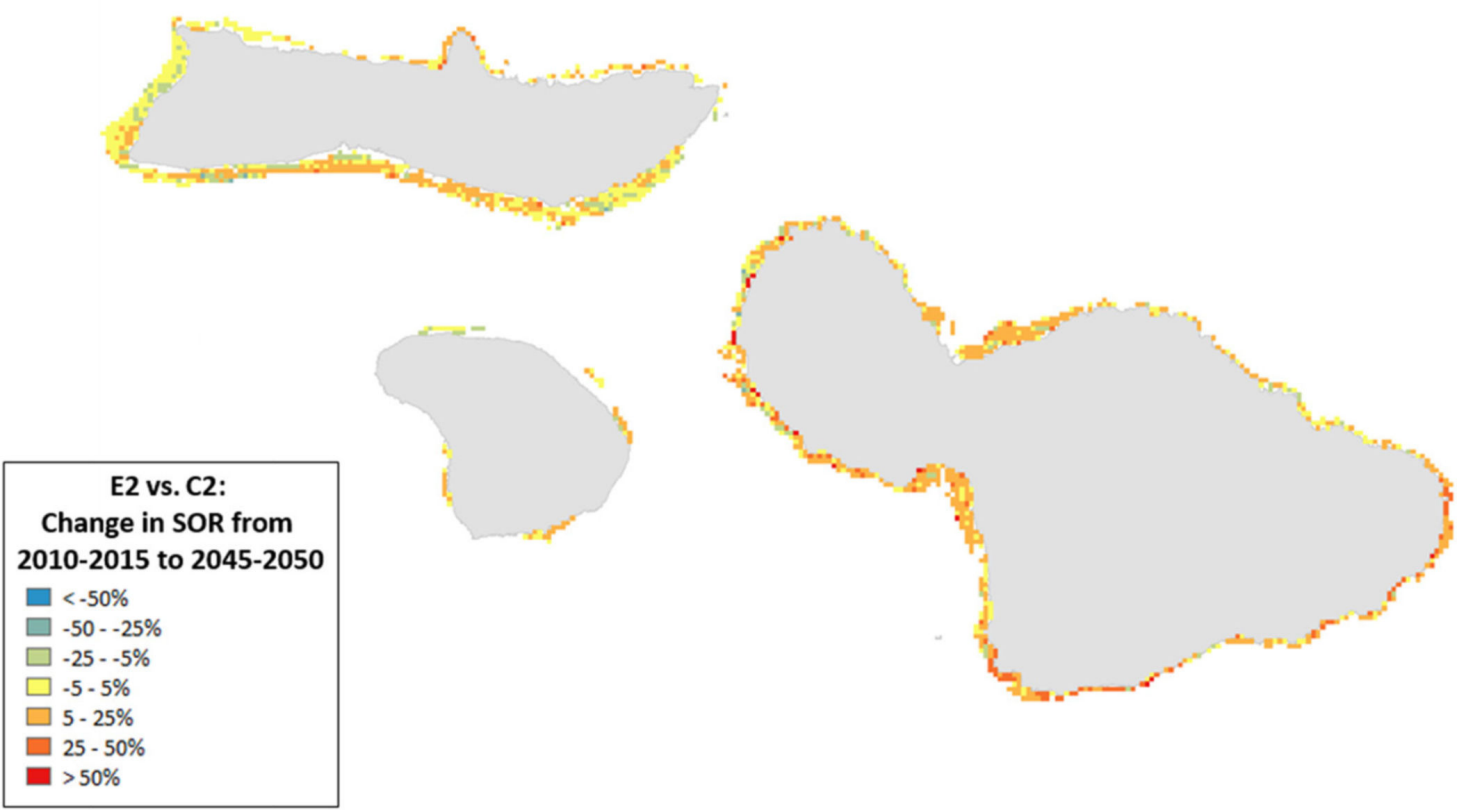
Ratio of the change in State of the Reef (2010–2050) for scenario E2 (high sediment mitigation, 30% MPAs) compared to C2 (Current Management) under less severe climate-related stressors. A value of 0 indicates no change from the outcome in 2050 under Current Management, values >0 indicate an improvement (or, more accurately, less of a decline from the Current Management), while values <0 indicate a worsening of the decline relative to Current Management.

Spatial variation in the results of management can provide insights into where to target local management. For example, the southern coastlines of Moloka’i and Maui showed declines for the State of the Reef and strict local management had little eﬀect (Figure 8). By contrast, under less severe climate change, local management improved State of the Reef along the same coastlines (Figure 9). However, there were also some places (designated in blue) where management exacerbated declines. Generally, management had a positive impact along most of the coastlines (red areas in Figures 8, 9).

**FIGURE 10 |:**
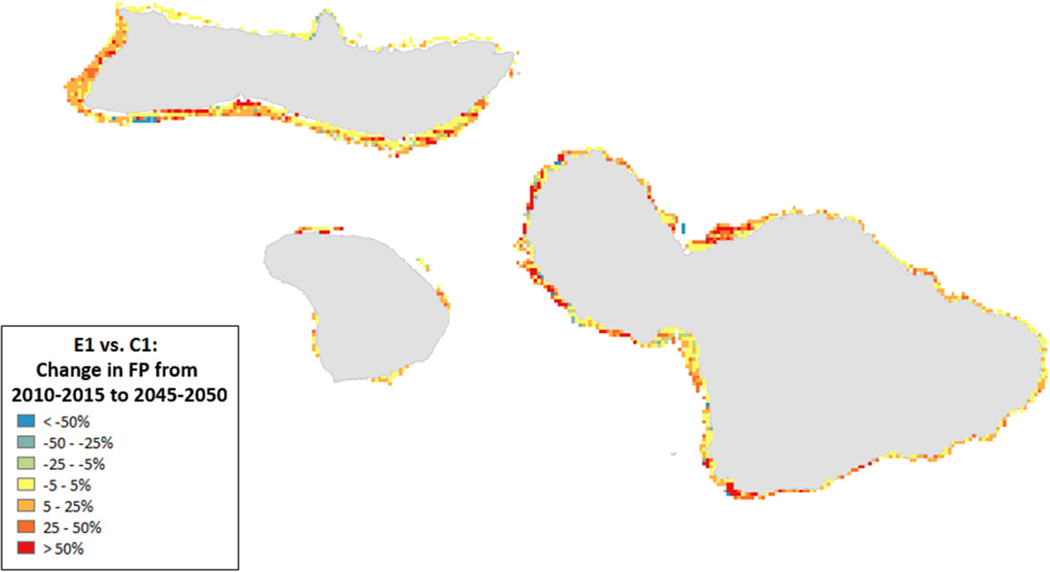
Ratio of change in Fisheries Production (2010–2050) for scenario E1 (high sediment mitigation, 30% MPAs) compared to C1 (Current Management) under severe climate-related stressors. A value of 0 indicates no change from the outcome in 2050 under Current Management, values >0 indicate an improvement (or, more accurately, less of a decline from the Current Management), while values <0 indicate a worsening of the decline relative to Current Management.

Spatial patterns in Fisheries Production showed improvement under all scenarios and along most coastlines but less so along the northern coastline of Moloka’i (we note that this area had relatively high biomass at the initialization of the scenario runs; Figure 10). Although in general many areas showed improvement, all coastlines also experienced losses; up to 100% in some places (Supplementary Figures S6). Management that included a 20% reduction in piscivore fish catches (Scenario F) tended to decrease overall Fisheries Production due to an increase in piscivores that preyed on herbivores (Supplementary Figures S6). At the same time, certain areas showed improvements with management partially due to spatial arrangement of the MPAs.

Trophic Integrity improved most along the southern coastline of Moloka’i and all around Maui (Supplementary Figures S6). As with the State of the Reef, the severity of climate change greatly influenced the results with much higher improvements under the less severe climate change scenarios.

### DISCUSSION

Marine resource managers are challenged with accounting for the cumulative eﬀects of local and global stressors on coral reef ecosystems and the valuable services reefs provide to society. However, environmental conditions and human use can result in considerable spatial variability of both reef fish biomass as well as benthic community (Williams et al., 2015; Cinner et al., 2016; Gorospe et al., 2018). Managers can use spatially-explicit decision-support tools to prioritize areas of high ecosystem service value that could benefit from action. Scenarios of future conditions can guide decision-makers as to which of these actions are robust to projected climate impacts.

We evaluated the impacts of land-based vs. marine-based management and local vs. global stressors by assessing the relationships between diﬀerent functional groups over time and under various management strategies and severities of climate-related stressors. As a metric for the eﬀectiveness of these diﬀerent strategies, four EPFs were used to model changes in ecosystem services; these ecosystem services best represented the economic interests of the State of Hawai’i pertaining to reef structure and resilience and reef-derived benefits.

Management of Hawai’i’s valuable nearshore areas in the face of local and global stressors will need to be adaptive to changing conditions. As more people choose to live in or visit Hawai’i, local stressors will continue to mount, sometimes in unexpected places or ways. Impacts from global climate change oﬀer another dimension of surprise. HIReefSim is an important tool to support adaptive local marine resource decision making. Our results suggest that policies are needed to mitigate local threats, and that these policies should consider future risks of impacts from climate-related stressors, such as changing hurricane patterns and coral bleaching, and likely also sea level rise. In 2016, the governor of Hawai’i pledged to “eﬀectively manage” 30% of the marine areas along the coastline by 2030, launching a multi-year marine spatial planning process. Key results from our analysis oﬀer a number of suggestions for adapting near-term management to long-term conditions. First of all, strict management of all local pressures (i.e., land-based and fisheries) is needed everywhere–not just across 30% of the nearshore area–to obtain the best results for reef state and trophic integrity under all climate change scenarios. Secondly, place matters. We found that spatial variation in the eﬀects of local management was particularly high for reef state and fisheries production, and moderate for trophic integrity and fisheries landings, suggesting that fine-tuning place-based management could improve outcomes.

### Does Local Management Matter in the Face of Climate-Related Stressors?

Yes, despite a decline in Trophic Integrity and State of the Reef EPFs, both these EPFs displayed a buﬀering eﬀect of strict management on the degradation of coral reefs, mostly under the less severe climate change scenarios. However, as a tradeoﬀ, Fisheries Landings decreased overall compared to today’s levels but the maximum decrease was just under 6% (Figure 6). Average Fisheries Production increased between 42% (scenario C2) and 38% (scenario D2) mostly due to the increases in herbivore biomass correlated with increases in turf algae. In absolute terms, local sediment control was particularly critical in slowing the decline of reefs under severe climate change. In these scenarios, coral cover was more prone to decline rapidly, which underscores both the importance of reducing greenhouse gases and implementing proactive, stringent management policies to mitigate declines in coral cover (Ortiz et al., 2014; Hughes et al., 2017). These results correspond to other modelling studies. Weijerman et al. (2015) showed that reefs in Guam would experience devastating declines under projected annual bleaching events despite local management directed at reduced land-based pollution and fishing. The same result was found for a Caribbean reef where, under the projected greenhouse gas emissions of a business-as-usual scenario, corals failed to recover from frequent bleaching events (Ortiz et al., 2014). Reducing sediments is not a complete solution given that nutrients and other contaminants of concern are still present and may have a very diﬀerent spatial signature. We focused on sediment impacts for this project based on the completeness and trustworthiness of available data layers, but future eﬀorts may consider the impacts of other sources of land-based pollution.

Herbivores appeared to fare well under climate-related stressors as coral cover declined and algal cover increased (Figures 6, 7). The strong correlation between algae and herbivores could be explained by the control of bottom-up, not top-down, mechanisms. A comparison of piscivore biomass in the populated Main Hawaiian Islands (MHI) with the non-fished Northwestern Hawaiian Islands (NWHI) shows that piscivore biomass was about 10–44 times higher in the NWHI (Williams et al., 2011). Even within the MHI, relatively remote and inaccessible locations had about five times the biomass of apex predators compared to more open areas (Williams et al., 2008). Thus, the low biomass of piscivores observed in the MHI could explain why top-down eﬀects may not be strong enough to explain some of the results, and suggests that strict fishery management is required to improve predation as an ecosystem function (Figure 5, Figure S5.7).

Alternatively, the increase in piscivores could be viewed as an undesired outcome since more piscivores led to elevated predation pressure on herbivores, reducing their biomass and the overall Fisheries Production (Figures 5–7). Ecologically, a higher trophic level (piscivores have a trophic level of 3.5–5 compared to a level of 2 for herbivores) of the fish community represents an ecosystem that is energetically more optimal and mature (Graham et al., 2017). Hence, for ecological reasons, one might want to strive to obtain a fish community with a high composition of piscivores. Economically, this also makes sense as in general piscivores sell for a higher price ($3.5–$5 per pound) compared to herbivores ($2–$3.5 per pound). Thus, although the results indicate a lower Fisheries Production under local management scenarios, this is not necessarily a bad thing; the Trophic Integrity of the Reef improved with more stringent management (E, F; Figure 7), and the reduced catch may not translate into large economic losses.

Wide-scale spatial variability across Maui Nui was projected for the EPFs, with declines of up to 100% across the northern shores of Maui and Moloka’i for Fisheries Production, and large declines in State of the Reef along southern and eastern shores (where the impacts of hurricanes were projected to be highest), highlighting the importance of identifying areas where management will likely be most successful.

### How Important Is It to Consider Both Land- and Marine-Based Threats in Local Management Action?

Eﬀectively managing the entire coral reef ecosystem requires the combination of both land-based and marine-based approaches. Land-based management was most beneficial for the benthic community, especially coral cover, whereas marine-based management not only decreased macroalgal cover, opening up substrate for reef calcifying CCA, but most notably decreased the downward trend in piscivore biomass (Figure 3). Of equal importance in evaluating the eﬀectiveness of marine-based management (MPAs) are the often-conflicting extraction and conservation objectives (Dichmont et al., 2013). If the objective of MPAs is to improve coral cover, MPA establishment proved less eﬀective than reducing land-based pollution (Figure 3). MPAs can enhance the resilience of coral reef ecosystems through trophic interactions (Mellin et al., 2016) or lead to small increases in coral cover (Graham et al., 2011), but reducing the main local stressors that drive coral decline directly (such as sediment inputs) is more eﬀective (ISRS, 2004) as the model results also showed. For maintaining or increasing key ecosystem functions under the projected impacts of climate-related stressors, no single management tool was eﬀective, but a combination of both land-based and marine-based management was needed as well as a reduction in greenhouse gas emissions (Figures 5– 7). These modeling results are corroborated by other studies (Dichmont et al., 2013; Weijerman et al., 2015; Arias-Gonzalez et al., 2017).

Random placement of no-take MPAs may not benefit the overall benthic community (Figure 3). For example, the 30% MPA designation in scenario B1 mitigated loss of fish biomass less than B2 for fish biomass, but improved the fate of corals (Figure 3). Placing MPAs in areas with currently the highest 10% of fish biomass and coral cover (B3) also increased the piscivore biomass but when (almost) doubling this area by making the current MPAs also no-take MPAs (B4), piscivore biomass did not double and catches stayed very similar (Figure 4). Likely, the areas which currently have the highest fish biomass are already somewhat exempt from high fishing pressure, either because they are diﬃcult to access or because only few people live close by, underscoring the importance of MPA placements. A separate issue is whether MPAs can achieve desired outcomes, as that also depends largely on strong governance (Cinner et al., 2016) and compliance (Gill et al., 2017).

## CONCLUSIONS

Overall, in the face of global threats, local management had mixed results for the ecosystem goods and services the reef provides. It abated the decline in Trophic Integrity of the reef and improved State of the Reef especially when both land-based and marine based approaches were combined but reducing greenhouse gas emissions is essential to avoid catastrophic loss of ecosystem services. Management that was more stringent required trading oﬀ Fisheries Production and Landings. By including extraction and reef resilience objectives in ecosystem goods and services, we provide a generalizable tool to clearly evaluate the tradeoﬀ of these conflicting goals under various management and climate-related stressors. Based on the limitations of the model structure, a cautionary interpretation of the model’s predicted long-term trajectory should be taken. Future work may want to improve the resource fish ratio, which we assumed to stay constant over the entire period. This assumption is unrealistic because fishers are likely to target specific species (e.g., jacks or other piscivores), causing community structure to shift. Furthermore, the results from the small number of MPA scenarios call for more thorough analysis, particularly with respect to their interaction with land-based pollution control. The model could also be used for evaluating the relative eﬀectiveness of diﬀerent fisheries management strategies, e.g., targeting diﬀerent trophic groups. A similar endeavor could focus on compliance within those MPAs to test the eﬀectiveness of restriction levels. Results of these improvements could greatly benefit the ongoing discussions of how to manage 30% of the coastline eﬀectively.

## Supplementary Material

sup1

## Figures and Tables

**TABLE 1 |:** Spatially-explicit stressors used to force scenario simulations in HIReefSim.

Variable	Variable description	Source

Fishing pressure (kg/km^2^/yr)	Annual average catch of reef fish (commercial, non-commercial shore-based, and non-commercial boat-based) per cell from past 10 years of records.	(Wedding et al., 2017)
Sediment input (kg/km^2^/yr)	Sediment plumes originating from stream mouths and coastal pour points.	(Wedding et al., 2017) (Supplementary Text S3)
Nutrient input (kg/km^2^/yr)	Nitrogen flux from onsite waste disposal systems (i.e., cesspools and septic tanks) and fertilizers.	(Wedding et al., 2017), Land use maps from 1920 & 2010 (Supplementary Text S3)
Hurricane zones	8 regions uniquely impacted by northwestern winter swells and tsunami waves from the southeast.	Dr. Bill Ward, NOAA National Weather Service, Pacific Region Headquarters

**TABLE 2 |:** Descriptions of 16 forecast simulations from 2010 to 2050.

Scenario type	Scenario name	Description	Primary parameters modification

No additional management, no climate-related stressors	CM: Current Management	Simulation of current management (~ 2% no-take MPAs and ~ 10% as MPA) under projected increase in human population with corresponding increased sediment and nutrient loads and fishing pressure.	See Supplementary Text S2 for base parameters

Land-based management, no climate-related stressors	A1 : High sediment mitigation	Sediment input reduced compared to	A1 : 0.38 * Current Management
	A2: Low sediment mitigation	Current situation for each scenario	A2: 0.94 * Current Management

Marine-based management, no climate-related stressors	B1_30	Additional MPAs on randomly selected reef areas comprising 30% of total reef area; new areas designated as no-take	All parameters set to Current Management values with fishing effort restricted according to size of MPAs
	B2_30	As B1_30 with a different set of randomly selected reef areas	
	B3_10	Additional MPAs created of areas encompassing the top 10% of coral cover and fish biomass; new areas designated as no-take	
	B4_20	Additional MPAs created as under B3_10 but encompassing top 20%, and current MPAs also designated as no-take	

No additional management, climate-related stressors	C1 : Current Management combined with high frequency of bleaching events C2: Current Management combined with low frequency of bleaching events	Severe and less severe climate-related scenarios. Both have hurricanes every 10 years. The severe (C1) scenario has increased bleaching-related coral mortality events every 2 years and the less severe scenario (C2) every 5 years. Both scenarios have no implementation of additional management strategies (Current Management).	• hfreq = 10; mean number of years between hurricanes • hdam_C = 0.49; factor by which coral cover is reduced during hurricane events • hdam_M = 0.49; factor by which macroalgal cover is reduced during hurricane event • cmfreq = 1 or 5; mean number of years between bleaching events • cm_C =0.3; factor by which coral cover is reduced during coral mortality events

Management and climate-related stressors	(D) Least effort sediment mitigation methods and MPA expansion under climate-related stressors	Combinations of fishery management B3_10 (top 10% coral cover and fish biomass are added as no-take MPAs) and land-based management A2 (low erosion mitigation efforts) with bleaching-related coral mortality events every 2 (D1) and 5 years (D2)	• Parameters hfreq, hdam_C, hdam_M, and cm_C as in scenarios C1 or C2 • 0.94 * CM sediment input (A2) • Additional 10% MPAs (B3_10)

Management and climate-related stressors	(E) High effort sediment mitigation methods and 30% MPA expansion under climate-related stressors	Combinations of fishery management B2_30 (30% randomly selected reef areas are added as no-take MPAs) and land-based management A1 (high erosion mitigation efforts) with bleaching-related coral mortality events every 2 (E1) and 5 years (E2)	• Parameters hfreq, hdam_C, hdam_M, and cm_C as in scenarios C1 or C2 • 0.38 * CM sediment input (A1) • Additional 30% MPAs as no-take (B2_30)

Management and climate-related stressors	(F) Strict management as under scenario G and 20% reduced piscivore fishing effort under climate-related stressors	As Scenario E with an additional 20% reduction in fishing effort of piscivores and bleaching-related coral mortality events every 2 (F1) and 5 years (F2)	• Parameters hfreq, hdam_C, hdam_M, and cm_C as in scenarios C1 or C2 • 0.38 * CM sediment input (A1) • Additional 30% MPAs as no-take (B2_30) • Reduced total piscivore fishing by 20%

Parameters are specified in Supplementary Text S2.

## References

[R1] Alvarez-Filip L, Dulvy NK, Gill JA, Côté IM, and Watkinson AR (2009). Flattening of caribbean coral reefs: region-wide declines in architectural complexity. Proc. R. Soc. B Biol. Sci. 276, 3019–3025. doi:10.1098/rspb.2009.0339 PMC2817220 19515663

[R2] Álvarez-Romero JG, Pressey RL, Ban NC, Vance-Borland K, Willer C, Klein CJ, (2011). Integrated land-sea conservation planning: the missing links. Annu. Rev. Ecol. Evol. Syst. 42, 381–409. doi:10.1146/annurev-ecolsys-102209-144702

[R3] Arias-Gonzalez JE, Fung T, Seymour RM, Garza-Perez JR, Acosta-Gonzalez G, Bozec Y-M, (2017). A coral-algal phase shift in mesoamerica not driven by changes in herbivorous fish abundance. PLoS ONE 12:e0174855. doi:10.1371/journal.pone.0174855 28445546 PMC5405933

[R4] Arkema KK, Abramson SC, and Dewsbury BM (2006). Marine ecosystem-based management: from characterization to implementation. Front. Ecol. Environ. 4, 525–532. doi:10.1890/1540-9295(2006)4[525:MEMFCT]2.0.CO;2

[R5] Bagstad KJ, Semmens DJ, Waage S, and Winthrop R. (2013). A comparative assessment of decision-support tools for ecosystem services quantification and valuation. Ecosyst. Serv. 5, 27–39. doi:10.1016/j.ecoser.2013.07.004

[R6] Barbier EB, Hacker SD, Kennedy C, Koch EW, Stier AC, and Silliman BR (2011). The value of estuarine and coastal ecosystem services. Ecol. Monogr. 81, 169–193. doi:10.1890/10-1510.1

[R7] Bellwood DR, Hughes TP, Folke C, and Nystrom M. (2004). Confronting the coral reef crisis. Nature 429, 827–833. doi:10.1038/nature02691 15215854

[R8] Brainard RE, Weijerman M, Eakin CMM, McElhany P, Miller MW, Patterson M, (2013). Incorporating climate and ocean change into extinction risk assessments for 82 coral species. Conserv. Biol. 27, 1169–1178. doi:10.1111/cobi.12171 24299083

[R9] Burke L, Reytar K, Spalding M, and Perry A. (2011). Reefs at Risk Revisited. Washington, DC: World Resources Institute.

[R10] Carlton JT, and Scanlon JA (1985). Progression and dispersal of an introduced alga: Codium fragile ssp. tomentosoides (Chlorophyta) on the Atlantic Coast of North America. Bot. Mar. 28:155. doi:10.1515/botm.1985.28.4.155

[R11] Cinner JE, Huchery C, MacNeil MA, Graham NAJ, McClanahan TR, Maina J, (2016). Bright spots among the world’s coral reefs. Nature 535, 416–419. doi:10.1038/nature18607 27309809

[R12] Compton JE, Harrison JA, Dennis RL, Greaver TL, Hill BH, Jordan SJ, (2011). Ecosystem services altered by human changes in the nitrogen cycle: a new perspective for US decision making. Ecol. Lett. 14, 804–815. doi:10.1111/j.1461-0248.2011.01631.x 21624028

[R13] Daily GC, and Matson PA (2008). Ecosystem services: from theory to implementation. Proc. Natl. Acad. Sci. U.S.A. 105, 9455–9456. doi:10.1073/pnas.0804960105 18621697 PMC2474530

[R14] DBEDT (2016). DBEDT 2014 Series. Popul. Econ. Proj. State Hawai’i to 2040. Available online at: http://dbedt.hawaii.gov/economic/economic-forecast/2045-long-range-forecast/ (Accessed July 12, 2016).

[R15] de Groot RS, Wilson MA, and Boumans RMJJ (2002). A typology for the classification, description and valuation of ecosystem functions, goods and services. Ecol. Econ. 41, 393–408. doi:10.1016/S0921-8009(02)00089-7

[R16] Delevaux JMS (2017). Data and Tools to Operationalize Ridge-to-Reef Management.Univ. of Hawaii at Manoa, Mgmt.Ph.D. dissertation. Dept. of Nat. Res. Envrin.

[R17] DeMartini EE, Anderson TW, Kenyon JC, Beets JP, and Friedlander AM (2010). Management implications of juvenile reef fish habitat preferences and coral susceptibility to stressors. Mar. Freshw. Res. 61, 532–540. doi:10.1071/MF09141

[R18] Dichmont CM, Ellis N, Bustamante RH, Deng R, Tickell S, Pascual R, (2013). Evaluating marine spatial closures with conflicting fisheries and conservation objectives. J. Appl. Ecol. 50, 1060–1070. doi:10.1111/1365-2664.12110

[R19] Dollar SJ, and Tribble GW (1993). Recurrent storm disturbance and recovery: a long-term study of coral communities in Hawaii. Coral Reefs 12, 223–233. doi:10.1007/BF00334481

[R20] Emanuel K. (2005). Increasing destructiveness of tropical cyclones over the past 30 years. Nature 436, 686–688. doi:10.1038/nature03906 16056221

[R21] Fung TC (2009). Local Scale Models of Coral Reef Ecosystems for Scenario Testing and Decision Support. Univ. Coll. London, Fac. Maths Phys. Sci. Ph.D. dissertation.

[R22] Gill DA, Mascia MB, Ahmadia GN, Glew L, Lester SE, Barnes M, (2017). Capacity shortfalls hinder the performance of marine protected areas globally. Nature 543, 665–669. doi:10.1038/nature21708 28329771

[R23] Gorospe KD, Donahue MJ, Heenan A, Gove JM, Williams ID, and Brainard RE (2018). Local biomass baselines and the recovery potential for Hawaiian coral reef fish communities. Front. Mar. Sci. 5:162. doi:10.3389/fmars.2018.00162

[R24] Graham NAJ, McClanahan TR, MacNeil MA, Wilson SK, Cinner JE, Huchery C, (2017). Human disruption of coral reef trophic structure. Curr. Biol. 27, 231–236. doi:10.1016/j.cub.2016.10.062 28089513

[R25] Graham NAJ, and Nash KL (2013). The importance of structural complexity in coral reef ecosystems. Coral Reefs 32, 315–326. doi:10.1007/s00338-012-0984-y

[R26] Graham NAJ, Nash KL, and Kool JT (2011). Coral reef recovery dynamics in a changing world. Coral Reefs 30, 283–294. doi:10.1007/s00338-010-0717-z

[R27] Gratwicke B, Speight MR, and Road SP (2005). The relationship between fish species richness, abundance and habitat complexity in a range of shallow tropical marine habitats. J. Fish Biol. 66, 650–667. doi:10.1111/j.0022-1112.2005.00629.x

[R28] Gurney GG, Melbourne-Thomas J, Geronimo RC, Aliño PM, and Johnson CR (2013). Modelling coral reef futures to inform management: can reducing local-scale stressors conserve reefs under climate change? PLoS ONE 8:e80137. doi:10.1371/2Fjournal.pone.0080137 24260347 PMC3832406

[R29] Heithaus MR, Frid A, Wirsing AJ, and Worm B. (2008). Predicting ecological consequences of marine top predator declines. Trends Ecol. Evol. 23, 202–210. doi:10.1016/j.tree.2008.01.003 18308421

[R30] Hoegh-Guldberg O. (1999). Climate change, coral bleaching and the future of the world’s coral reefs. Mar. Freshw. Res. 50, 839–866.

[R31] Hoegh-Guldberg O, Andréfouët S, Fabricius KE, Lough JM, Marshall PA, and Pratchett MS (2011). “Vulnerability of coral reefs in the tropical Pacific to climate change,” in Vulnerability of Tropical Pacific Fisheries and Aquaculture to Climate Change, eds Bell J, Johnson J, and Hobday A(Noumea, New Caledonia: Secretariat of the Pacific Community), 251–296.

[R32] Hughes T, Kerry J, Álvarez-Noriega M, Álvarez-Romero J, Anderson K, Baird A, (2017). Global warming and recurrent mass bleaching of corals. Nature 543, 373–377. doi:10.1038/nature21707 28300113

[R33] Hughes TP, Graham NAJ, Jackson JBC, Mumby PJ, and Steneck RS (2010). Rising to the challenge of sustaining coral reef resilience. Trends Ecol. Evol. 25, 633–642. doi:10.1016/j.tree.2010.07.011 20800316

[R34] Hughes TP, Rodrigues MJ, Bellwood DR, Ceccarelli D, Hoegh-Guldberg O, McCook L, (2007). Phase shifts, herbivory, and the resilience of coral reefs to climate change. Curr. Biol. 17, 360–365. doi:10.1016/j.cub.2006.12.049 17291763

[R35] Hulme P. (2005). Adapting to climate change: is there scope for ecological management in the face of a global threat? J. Appl. Ecol. 42, 784–794. doi:10.1111/j.1365-2664.2005.01082.x

[R36] ISRS (2004). The Effects of Terrestrial Runoff of Sediments, Nutrients and Other Pollutants on Coral Reefs. Briefing Paper 3, International Society for Reef Studies.

[R37] Jokiel PL, and Coles SL (1990). Response of Hawaiian and other Indo-Pacific reef corals to elevated temperature. Coral Reefs 8, 155–162. doi:10.1007/BF00265006

[R38] Kapur MR, and Franklin EC (2017). Simulating future climate impacts on tropical fisheries: are contemporary spatial fishery management strategies sufficient? Can. J. Fish. Aquat. Sci. 74, 1974–1989. doi:10.1139/cjfas-2016-0200

[R39] Kennedy EV, Perry CT, Halloran PR, Iglesias-Prieto R, Schönberg CHL, Wisshak M, (2013). Avoiding coral reef functional collapse requires local and global action. Curr. Biol. 23, 912–918. doi:10.1016/j.cub.2013.04.020 23664976

[R40] Levin PS, Kelble CR, Shuford RL, Ainsworth C, Dunsmore R, Fogarty MJ, (2013). Guidance for implementation of integrated ecosystem assessments: a US perspective. ICES J. Mar. Sci. 112, 1198–1204. doi:10.1093/icesjms/fst112

[R41] Liu X, and Xing Y. (2012). Qualitative analysis for a predator prey system with holling type III functional response and prey refuge. Discret. Dyn. Nat. Soc. 2012:678957. doi:10.1155/2012/678957

[R42] Maynard J, van Hooidonk R, Eakin CM, Puotinen M, Garren M, Williams G, (2015b). Projections of Climate Conditions that Increase Coral Disease Susceptibility and Pathogen Abundance and Virulence. Nat. Clim. Chang. 5, 1–8. doi:10.1038/NCLIMATE.2625

[R43] Maynard JA, McKagan S, Raymundo L, Johnson S, Ahmadia GN, Johnston L, (2015a). Assessing relative resilience potential of coral reefs to inform management. Biol. Conserv. 192, 109–119. doi:10.1016/j.biocon.2015.09.001

[R44] McClanahan TR, Graham NAJ, and Darling ES (2014). Coral reefs in a crystal ball: predicting the future from the vulnerability of corals and reef fishes to multiple stressors. Curr. Opin. Environ. Sustain. 7, 59–64. doi:10.1016/j.cosust.2013.11.028

[R45] McCoy KS, Williams ID, Friedlander AM, Hongguang M, Teneva LT, and Kittinger JN (2018). Estimating nearshore coral reef-associated fisheries production from the main Hawaiian Islands using commercial and non-commercial data. PLoS ONE 13:e0195840. doi:10.1371/journal.pone.0195840 29659616 PMC5901996

[R46] Melbourne-Thomas J, Johnson CR, Fung T, Seymour RM, Chérubin LM, Arias-González JE, (2011a). Regional-scale scenario modeling for coral reefs: a decision support tool to inform management of a complex system. Ecol. Appl. 21, 1380–1398. doi:10.1890/09-1564.1 21774437

[R47] Melbourne-Thomas J, Johnson CR, Perez P, Eustache J, Fulton EA, and Cleland D. (2011b). Coupling biophysical and socioeconomic models for coral reef systems in quintana roo, Mexican Caribbean. Ecol. Soc. 16:23.

[R48] Mellin C, MacNeil MA, Cheal AJ, Emslie MJ, Julian Caley M, MacNeiil MA, (2016). Marine protected areas increase resilience among coral reef communities. Ecol. Lett. 19, 629–637. doi:10.1111/ele.12598 27038889

[R49] Millennium Ecosystem Assessment (2005). Ecosystems and Human Well-Being: Current State and Trends. eds Hassan R and Scholes R Washington, DC: Island Press.

[R50] Moberg F, and Folke C. (1999). Ecological goods and services of coral reef ecosystems. Ecol. Econ. 29, 215–233. doi:10.1016/S0921-8009(99)00009-9

[R51] Murakami H, Wang B, Li T, and Kitoh A. (2013). Projected increase in tropical cyclones near Hawaii. Nat. Clim. Chang. 3, 749–754. doi:10.1038/nclimate1890

[R52] Nelson E, Mendoza G, Regetz J, Polasky S, Tallis H, Cameron DR, (2009). Modeling multiple ecosystem services, biodiversity conservation, commodity production, and tradeoffs at landscape scales. Front. Ecol. Environ. 7, 4–11. doi:10.1890/080023

[R53] Orlando JL, and Yee SH (2017). Linking terrigenous sediment delivery to declines in coral reef ecosystem services. Estuaries Coasts 40, 359–375. doi:10.1007/s12237-016-0167-0 30123101 PMC6093629

[R54] Ortiz JC, Bozec Y-M, Wolff NH, Doropoulos C, and Mumby PJ (2014). Global disparity in the ecological benefits of reducing carbon emissions for coral reefs. Nat. Clim. Chang. 4, 1090–1094. doi:10.1038/nclimate2439

[R55] Peters GP, Le Quér,é C, Andrew RM, Canadell JG, Friedlingstein P, Ilyina T, (2017). Towards real-time verification of CO2 emissions. Nat. Clim. Chang. 7, 848–850. doi:10.1038/s41558-017-0013-9

[R56] Pörtner HO, Langenbuch M, and Michaelidis B. (2005). Synergistic effects of temperature extremes, hypoxia, and increases in CO_2_ on marine animals: from Earth history to global change. J. Geophys. Res. Ocean. 110:C09S10. doi:10.1029/2004JC002561

[R57] Principe PP, Bradley P, Yee SH, Fisher WS, Johnson ED, Allen P, (2012). Quantifying Coral Reef Ecosystem Services. Washighton, DC: U.S. Environmental Protection Agency.

[R58] Prouty NG, Storlazzi CD, Mccutcheon AL, and Jenson JW (2014). Historic impact of watershed change and sedimentation to reefs along west-central Guam. Coral Reefs 33, 733–749. doi:10.1007/s00338-014-1166-x

[R59] Rogers A, Blanchard JL, and Mumby PJ (2014). Vulnerability of coral reef fisheries to a loss of structural complexity. Curr. Biol. 24, 1000–1005. doi:10.1016/j.cub.2014.03.026 24746794

[R60] Ruttenberg BI, Hamilton SL, Walsh SM, Donovan MK, Friedlander A, DeMartini E, (2011). Predator-induced demographic shifts in coral reef fish assemblages. PLoS ONE 6:e21062. doi:10.1371/journal.pone.0021062 21698165 PMC3116880

[R61] Selig ER, Casey KS, and Bruno JF (2012). Temperature-driven coral decline: the role of marine protected areas. Glob. Chang. Biol. 18, 1561–1570. doi:10.1111/j.1365-2486.2012.02658.x

[R62] Spalding M, Burke L, Wood SA, Ashpole J, Hutchison J, and zu Ermgassen P. (2017). Mapping the global value and distribution of coral reef tourism. Mar. Policy 82, 104–113. doi:10.1016/j.marpol.2017.05.014

[R63] Stamoulis KA, Poti M, Delevaux JMS, Donovan MK, Friedlander AM, and Kendall MS (2016). “4. Fishes - Reef Fish,” in Marine Biogeographic Assessment of the Main Hawaiian Islands, eds. Costa B and Kendall MS (Silver Spring, MD: OCS Study BOEM and NOAA Technical Memorandum NOS NCCOS 214), 156–196.

[R64] Thompson A, and Dolman A. (2010). Coral bleaching: one disturbance too many for near-shore reefs of the Great Barrier Reef. Coral Reefs 29, 637–648. doi:10.1007/s00338-009-0562-0

[R65] Van Beukering PJH, and Cesar HSJ (2004). Ecological Economic modeling of coral reefs: evaluating tourist overuse at hanauma bay and algae blooms at the kihei coast. Pacific Sci. 58, 243–260. doi:10.1353/psc.2004.0012

[R66] Van Hooidonk R, Maynard JA, and Planes S. (2013). Temporary refugia for coral reefs in a warming world. Nat. Clim. Chang. 3, 508–511. doi:10.1038/nclimate1829

[R67] Van Hooidonk RJ, Maynard J, Tamelander J, Gove J, Ahmadia G, Raymundo L, (2016). Local-scale projections of coral reef futures and implications of the paris agreement. Sci. Rep. 6:39666. doi:10.1038/srep39666 28000782 PMC5175274

[R68] Wainger L, and Boyd J. (2009). “Valuing ecosystem services,” in Ecosystem-Based Management for the Oceans, eds McLeod K and Leslie H (Washington, DC: Island Press), 99–114.

[R69] Wedding LM, Lecky JH, Gove J, Walecka HR, Donovan MK, Williams GJ, (2017). Advancing the integration of spatial data to map human and natural drivers on coral reefs. PLoS ONE 13:e0189792. doi:10.1371/journal.pone.0189792 PMC5832214 29494613

[R70] Weijerman M, Fulton EA, and Brainard RE (2016). Management strategy evaluation applied to coral reef ecosystems in support of ecosystem-based management. PLoS ONE 11:e0152577. doi:10.1371/journal.pone.01 27023183 PMC4811577

[R71] Weijerman M, Fulton EA, Kaplan IC, Gorton R, Leemans R, Mooij WM, (2015). An integrated coral reef ecosystem model to support resource management under a changing climate. PLoS ONE 10:e0144165. doi:10.1371/journal.pone.0144165 26672983 PMC4682628

[R72] Williams ID, Baum JK, Heenan A, Hanson KM, Nadon MO, and Brainard RE (2015). Human, oceanographic and habitat drivers of central and western Pacific coral reef fish assemblages. PLoS ONE 10:e0120516. doi:10.1371/journal.pone.0120516 25831196 PMC4382026

[R73] Williams ID, Richards BM, Sandin SA, Baum JK, Schroeder RE, Nadon MO, (2011). Differences in reef fish assemblages between populated and remote reefs spanning multiple archipelagos across the central and western Pacific. J. Mar. Biol. 2011:826234. doi:10.1155/2011/826234

[R74] Williams ID, Walsh WJ, Schroeder RE, Friedlander AM, Richards BL, and Stamoulis KA (2008). Assessing the importance of fishing impacts on Hawaiian coral reef fish assemblages along regional-scale human population gradients. Environ. Conserv. 35, 261–272. doi:10.1017/S0376892908004876

[R75] Wren JLK, and Kobayashi DR (2016). Exploration of the “larval pool”: development and ground-truthing of a larval transport model off leeward Hawai’i. PeerJ. 4:e1636. doi:10.7717/peerj.1636 26855873 PMC4741072

[R76] Yee SH, Dittmar JA, and Oliver LM (2014). Comparison of methods for quantifying reef ecosystem services: a case study mapping services for St. Croix, USVI. Ecosyst. Serv. 8, 1–15. doi:10.1016/j.ecoser.2014.01.001

