# Supplementary material for: Managing Local Stressors for Coral Reef Condition and Ecosystem Services Delivery Under Climate Scenarios
Source: Front Mar Sci. Author manuscript; Available in PMC 2021 Jun 11. (PMC8193846; doi:10.3389/fmars.2018.00425)
Supplement: sup1 [file NIHMS1535775-supplement-sup1.docx]

Supplementary Material

Managing local stressors for coral reef condition and ecosystem services delivery under climate scenarios

**Mariska Weijerman^*^, Lindsay Veazey, Susan Yee, Kellie Vaché, Jade Delevaux, Mary Donovan, Kim Falinski, Joey Lecky, Kirsten L.L. Oleson**

*** Correspondence:** Mariska Weijerman: Mariska.weijerman@noaa.gov

# Supplementary Table S1: Results of predicted models using Boosted Regression Trees for input variables used in HIReefSim.

| Response Variable | Tree Complexity | Learning Rate | Bag Fraction | No. of Trees | CV Mean % Deviance Explained | Test % Deviance Explained | Root Mean Square Error |
| --- | --- | --- | --- | --- | --- | --- | --- |
| Herbivore biomass (g m^-2^) | 10 | 0.01 | 0.5 | 500 | 32.06 | 33.03 | 61.97 |
| Piscivore biomass (g m^-2^) | 5 | 0.001 | 0.75 | 2700 | 8.50 | 6.36 | 1.25 |
| Resource fish biomass (g m^-2^) | 3 | 0.01 | 0.75 | 1750 | 32.01 | 30.57 | 108.22 |
| Fish Richness | 10 | 0.001 | 0.5 | 5150 | 47.04 | 49.69 | 7.67 |
| Coral cover (%) | 5 | 0.005 | 0.75 | 4450 | 60.02 | 65.43 | 13.41 |
| Crustose coralline algae cover (%) | 5 | 0.01 | 0.75 | 950 | 25.08 | 29.84 | 6.44 |
| Macroalgal cover (%) | 10 | 0.01 | 0.75 | 700 | 45.35 | 40.25 | 11.61 |
| Turf cover (%) | 10 | 0.001 | 0.75 | 4150 | 46.4 | 52.27 | 23.32 |

# Supplementary Text S2: Model algorithms and parameter estimation

A detailed evaluation of equilibrium behavior and parameter sensitivity of the local-scale system of equations for an analogous, continuous-time (differential equations) system is provided by Fung (2009 and for a discrete-time system as used in CORSET by Melbourne-Thomas et al (2011). The model uses seven algorithms to represent the changes in biomass and cover of the seven functional groups: brooding corals (*C_b_*), spawning corals (*C_s_*), macroturf algae (*T*), macroalgae (*M*), epilithic algal communities (which we use as crustose coralline algae, CCA) (*E*), herbivores (*H*), and piscivores (*P*). Following the methods outlined in Fung (2009) and using data specific to Hawaiʻi where possible and otherwise from reefs in the Indo-Pacific, we derived parameter ranges for reefs in the Maui Nui complex. Some values are ranges based on empirical observations. During model calibration we examined model sensitivity to these ranges and selected the value that best described past dynamics. For a few parameters related to fish growth (i.e.,g_T_, g_M_, μ_M_, μ_T_, μ_E_, i_PH_, and g_P_) the model proved to be sensitive and we selected values from that range at random, i.e. assuming a uniform distribution based on a lack of information regarding the exact shape of distributions for these ecological parameters.

Dynamic equations used in the model are:

Brooding corals:

$$\frac{dC_{b}}{dt}=r_{C}\left( 1-\beta_{M}M \right)\left( E+\alpha_{C}T \right)C_{b}-d_{C}C_{b}-\gamma_{MC}r_{M}MC_{b}+I_{C_{b}}(E+\varepsilon_{C}T)$$

growth mortality macroalgal overgrowth recruitment

Spawning corals:

$$\frac{dC_{s}}{dt}=r_{C}\left( 1-\beta_{M}M \right)\left( E+\alpha_{C}T \right)C_{s}-d_{C}C_{s}-\gamma_{MC}r_{M}MC_{s}+I_{C_{s}}(E+\varepsilon_{C}T)$$

growth mortality macroalgal overgrowth recruitment

Macroturf:

$$\frac{dT}{dt}=\zeta T\left( 1-\theta\right)E-g_{T}\theta T-r_{C}\alpha_{C}\left( 1-\beta_{M}M \right)CT-\gamma_{MT}r_{M}MT-\varepsilon_{C}(I_{C_{b}}+I_{C_{s}})T$$

growth grazing coral overgrowth macroalgal overgrowth coral recruits

Macroalgae:

$$\frac{dM}{dt}=r_{M}ME-g_{M}\theta M+\gamma_{MC}r_{M}MC+\gamma_{MT}r_{M}MT$$

growth over EAC grazing growth over coral growth over turf

Epilithic algae:

$$E=1-C_{b}-C_{s}- T-M$$

Herbivorous fish:

growth mortality predation

$$\frac{dH}{dt}=\theta_{H}\left( g_{M}{M\mu}_{M}+g_{T}T\mu_{T}+\zeta_{T}E\mu_{E} \right)-d_{H}H-g_{P}P\left( \frac{H^{2}}{{i^{2}}_{PH}+H^{2}} \right)$$

growth mortality predation

$$-\rho_{H}f\left( \frac{H}{i_{FH}+H} \right)+l_{H}$$

fishing recruitment

Piscivorous fish:

$$\frac{dP}{dt}=r_{P}g_{P}\left( \frac{H^{2}}{{i^{2}}_{PH}+H^{2}} \right)-d_{P}P-\rho_{P}f\left( \frac{P}{i_{FP}+P} \right)+l_{H}$$

growth mortality fishing recruitment

The consistent patterns of larval dispersal, along with the similar paths that drifters followed over multiple deployments, suggest that the temporally-limited drifter trajectories are a good model for initial (< 5 days) dispersal patterns off west Maui during the summer months when many of the Hawaiian corals spawn (Storlazzi et al., 2006). We modeled recruitment rates for spawning corals and fish using a connectivity matrix based on the 50 m depth layer of the Hybrid Coordinate Ocean Model (HYCOM, <https://hycom.org/hycom>) parameterized for Hawaiʻi and a pelagic larval duration of 45 days for fish and corals (Wren and Kobayashi, 2016). Recruitment for brooders is localized, i.e., through self-seeding.

There are 69 species of corals identified in Hawaiʻi (Veron and Stafford-Smith, 2002) with taxonomic uncertainty about some species (Forsman et al., 2010). Of these species, only a handful dominate the benthos (88%) and are important reef-building species. These prominent hermatypic corals include *Porites lobata*, *Montipora* spp., *Pocillopora* *meandrina*, and *Porites compressa*. Three-quarters of the coral species in Hawaiʻi are considered spawners based on a sample selection of 25 species (Kolinski, 2007). However, all dominant coral species in Hawaiʻi are spawners (e.g., *Montipora* spp., *Porites* spp.), and of the cumulative 98.5% coral species comprising the benthos, only 3.1% are brooders (PIFSC ESD unpublished data). Examples of species that are brooders include *Pocillopora damicornis*, *Porites lichen*, and *Cyphastrea ocellina* (Kolinski, 2007; Richmond and Hunter, 1990). To represent this dichotomy, we separated coral cover into 97% spawners and 3% brooders. For coral related parameters, we took the weighted mean of 95% of the cumulative total observed species.

Algal cover in Maui Nui has increased substantially over the last several decades, especially along the west coast of Maui due to the proliferation of invasive algae (Smith, 2006), which has prompted conservation action by the state of Hawaiʻi (Sparks et al., 2011). Algae were grouped in macroalgae (>1cm), turf algae (<1 cm) and crustose coralline algae (CCA). CCA was modeled as 1-other benthic cover groups.

CORSET differentiates between large (> 60 cm) and small piscivores and allows for urchin biomass input (Fung, 2009). No reliable urchin data were available for Hawai’i, so this group was omitted in the HIReefSim model. Additionally, data were not sufficient to group piscivores by size, so all piscivores were grouped together. Estimated HIReefSim parameter values based on the maximum herbivorous fish biomass differ substantially with values from Fung (Fung, 2009). Fung used the maximum values found on a biomass-rich New Caledonian reef (maximum total biomass 447 g/m^2^) which contrasts with total fish biomass observed across Maui Nui (36 g/m^2^ around Molokaʻi; PIFSC ESD visual survey data 2010 – 2015). Reefs across Hawaiʻi are heavily influenced by human population density and likely do not display their potential maximum biomass levels (Williams et al., 2015). We used the predicted biomass values for Maui Nui (66 g/m^2^) in the absence of human pressures (Williams et al., 2015). We assumed that 45% of total biomass was herbivorous fish based on the species composition observed during PIFSC-ESD visual surveys conducted from 2010 to 2015. This estimation yielded a maximum herbivore biomass of 30 g/m^2^, which is still much lower than the New Caledonian value, but grounded to the location of interest. For fish related parameter values we used weighted means of the various functional groups (e.g. browsers, grazers, small-bodied parrotfishes and large-bodies parrotfishes).

Parameters used are summarized in Table S2. For comparison, the values calculated by Melbourne-Thomas et al. (2011b) for the Indo-Pacific are also included.

**Table S2.** Parameter definitions and values derived for Maui Nui, Hawaiʻi. Values were derived following the methods outlined in Fung (2009) and adapted for Hawaiʻi where data were available. Benthic functional groups are represented by proportional cover and consumer groups by biomass (kg/km^2^). See Fung (2009) for details on mathematical derivations of parameter restrictions.

| **Parameter** | **Description** | **Indo-Pacific** | **Maui-Nui** | **Source** |
| --- | --- | --- | --- | --- |
| r_C_ | The growth rate of existing coral over grazed EAC (*r_C_*) | 0.1 – 0.2 yr^-1^ | 0.06 – 0.16 yr^-1^ | (Minton and Ph, 2013), PIFSC-ESD unpublished data |
| α_C_ | The growth rate of coral over macroturf, relative to its growth over grazed EAC (*α_C_*) | 0 – 1 | 0 – 1 | (Fung, 2009; McCook, 2001) |
| d_C_ | The background mortality rate of brooding and spawning corals (*d_C_*) | 0.02 – 0.1 yr^-1^ | 0.02 – 0.3 yr^-1^ | (Box and Mumby, 2007; Kolinski, 2007; Koop et al., 2001) |
| ε_C_ | The recruitment rate of coral onto macroturf, relative to the rate onto grazed EAC (*ε_C_*) | 0.05 – 0.15 | 0.1 – 0.4 | (Birrell et al., 2005; Box and Mumby, 2007) |
| ξ_Cb_ | The recruitment rates of brooding and spawning corals onto grazed EAC (ξ_Cb_, ξ_Cs_) | Modeled using connectivity matrices | | (Wren and Kobayashi, 2016) |
| ξ_Cs_ |  |  |  |  |
| *ζ*_T_ | The rate at which grazed EAC grows into macroturf (*ζ_T_*) | 2 – 20 yr^-1^ | 3 – 21 yr^-1^ | (Birkeland, 1977; Klumpp and McKinnon, 1992; Smith et al., 2001) |
| g_T_ | The maximum rate at which existing macroturf is grazed T down (*g_T_* ) | 5 – 15 yr^-1^ | 5 – 15 yr^-1^ | (Melbourne-Thomas et al., 2011b) |
| β_M_ | Coral growth is inhibited by the presence of nearby macroalgae and this is represented as depression of r_C_ by the factor (1 – *β_M_*) | 0.2 – 0.3 yr^-1^ | 0 – 0.3 yr^-1^ | (Lirman, 2001; McCook, 2001; Smith, 2006) |
| r_M_ | The growth rate of macroalgae over grazed EAC (*r_M_*) | 0.05 – 0.4 yr^-1^ | 0.01 – 0.5 yr^-1^ | (Smith et al., 2001) |
| g_M_ | The maximum rate (per unit of grazing pressure θ) at which existing macroalgae is grazed down (*g_M_*) | 0.01.g_T_ – 1 g_T_ yr^-1^ | 0.11 g_T_ – 1 g_T_ yr^-1^ | 11% of herbivorous fish eat macroalgae (CREP unpublished data) |
| *ϒ*_MC_ | The growth rate of macroalgae over coral, relative to its growth over grazed EAC (*ϒ_MC_*) | 0 – 0.9 | 0 – 0.9 | McCook et al (McCook, 2001) (0-1); Smith et al. (Smith, 2006) (0.3); Fung (Fung, 2009) (0 – 0.9) |
| *ϒ*_MT_ | The growth rate of macroalgae over turf, relative to its growth over grazed EAC (*ϒ_MT_*) | 0 – 0.9 | 0 – 0.9 | Due to paucity of data assuming same range as for macroalgae |
| d_H_ | The mortality rate of herbivorous fish from all factors other than predation by piscivorous fish and fishing (*d_H_*) | d_Hmin_ – 2 yr^-1^ | 0.2 – 1.1 yr^-1^ | Mortality values weighted by biomass. Fishbase.org, PIFSC-ESD unpublished data |
| ξ_H_ | The recruitment rate of herbivorous fish (ξ_H_) | Modeled using connectivity matrices | | (Wren and Kobayashi, 2016) |
| λ_H_ | A parameter that determines the competitiveness of herbivorous fish relative to urchins (λH) | 0.8 | 0.5-1 | (Fung, 2009) |
| ϕ_P_ | A constant parameter that determines how quickly small piscivorous fish biomass becomes large piscivorous fish biomass due to predation and subsequent growth (*ϕ_P_*) | 0 – 10 | 0 | Since we model piscivores as one group we set this to zero to retain all piscivores in the same group |
| r_P_ | The proportion of consumed biomass which is used for somatic growth, for piscivorous fish (*r_P_* ) | 0.07 – 0.1 | 0.07 –0.25 | (Weijerman et al., 2013) |
| g_P_ | The maximum predation rate of piscivorous fish on herbivorous fish (*g_P_* ) | 1 – 2 yr^-1^ | 0.9 – 1.1 yr^-1^ | (Weijerman et al., 2013) |
| d_P_ | The mortality rate of piscivorous fish from all factors other than predation and fishing (*d_P_*) | 0.2 – 3 yr^-1^ | 0.2 – 0.6 yr^-1^ | Mortality values ([www.fishbase.org](http://www.fishbase.org)) for piscivorous fish weighted by biomass |
| ξ_P_ | The recruitment rate of herbivorous fish (*ξ_P_*)* | Modeled using connectivity matrices | | (Wren and Kobayashi, 2016) |
| μ_M_ | The herbivorous fish biomass accumulated from grazing on 100% cover of macroalgae (*μ_M_*) | 900 – 5 x 10^5^ kg.km^-2^.yr^-1^ | 800 – 9x10^4^ kg.km^-2^.yr^-1^ | PIFSC-ESD unpublished data |
| μ_T_ | The herbivorous fish biomass accumulated from grazing on 100% cover of macroturf (*μ_T_*) | 900 – 5 x 10^5^ kg.km^-2^.yr^-1^ | 800 – 9x10^4^ kg.km^-2^.yr^-1^ | PIFSC-ESD unpublished data |
| μ_E_ | The herbivorous fish biomass accumulated from grazing on 100% cover of EAC (*μ_E_*) | 900 – 5 x 10^5^ kg.km^-2^.yr^-1^ | 800 – 9x10^4^ kg.km^-2^.yr^-1^ | PIFSC-ESD unpublished data |
| i_H_ | A parameter that measures the inaccessibility of algae (macroturf and macroalgae) to herbivorous fish grazing (*i_H_*) | 1 x 10^3^ – 4 x 10^5^ kg/km^2^ | 1 x 10^3^ – 4 x 10^5^ kg/km^2^ | PIFSC-ESD unpublished data |
| i_PH_ | A parameter that measures the inaccessibility of herbivorous fish to predation by piscivorous fish (*i_PH_*) | 7 x 10^3^ – 1 x 10^4^ kg/km^2^ | 7.5 x 10^2^ – 1.5 x 10^3^ kg/km^2^ | PIFSC-ESD unpublished data |
| i_FH_ | A parameter that measures the inaccessibility of herbivorous fish to fishers (*i_FH_*) | 7 x 10^2^ – 1 x 10^3^ kg/km^2^ | 80 – 200 kg/km^2^ | PIFSC-ESD unpublished data |
| i_FP_ | A parameter that measures the inaccessibility of piscivorous fish to fishers (*i_FP_*) | 7 x 10^2^ – 1 x 10^3^ kg/km^2^ | 80 – 200 kg/km^2^ | PIFSC-ESD unpublished data |
| *f* | The maximum fishing biomass which can be caught (*f*) | 0 – 2 x 10^4^ kg.km^-2^.yr^-1^ | 0 – 940 kg.km^-2^.yr^-1^ | (Weijerman et al., 2013) |
| ρ_H_, ρ_P_ | The proportion of total fishing pressure *f* which acts on herbivorous fish (*ρ_H_* + *ρ_P_* = 1) | 0 – 1 | 0 – 1 | (Melbourne-Thomas et al., 2011a) |
| l_C_ | Brooding and spawning coral larval production (at 100% cover) | 6 x 10^9^ – 9 x 10^9^ kg.km^-2^.yr^-1^ | 6 x 10^9^ – 9 x 10^9^ kg.km^-2^.yr^-1^ | (Melbourne-Thomas et al., 2011a) |
| l_H_ | Herbivorous fish larval production | 4 x 10^3^ – 1 x 10^4^ kg.km^-2^.yr^-1^ | 4 x 10^3^ – 1 x 10^4^ kg.km^-2^.yr^-1^ | (Melbourne-Thomas et al., 2011a) |
| l_P_ | Piscivorous fish larval production | 1 x 10^2^ – 6 x 10^2^ kg.km^-2^.yr^-1^ | 1 x 10^2^ – 6 x 10^2^ kg.km^-2^.yr^-1^ | (Melbourne-Thomas et al., 2011a) |
| d_larvCb_ | The pre-settlement mortality rate for brooded coral larvae | 0.6 – 0.99 | 0.6 – 0.99 | (Melbourne-Thomas et al., 2011a) |
| d_recC_ | Mortality of brooding and spawning coral recruits during the first year following settlement | 0.2 – 0.7 yr^-1^ | 0.2 – 0.7 yr^-1^ | (Melbourne-Thomas et al., 2011a) |
| d_recF_ | Mortality of fish recruits during the first year following settlement^Ξ^ | 0.2 yr^-1^ | 0.2 yr^-1^ | (Melbourne-Thomas et al., 2011a) |
| d_recU_ | Mortality of urchin recruits during the first year following settlement^Ξ^ | 0.2 yr^-1^ | 0.2 yr^-1^ | (Melbourne-Thomas et al., 2011a) |
| area_C_ | The area of 1 year old coral recruits | 2.18 cm^2^ | 2.18 cm^2^ | (Melbourne-Thomas et al., 2011a) |
| b_recH_ | The biomass of 1 year old herbivorous fish recruits | 80 g | 80 g | (Melbourne-Thomas et al., 2011a) |
| b_recP_ | The biomass of 1yr old piscivorous fish recruits | 150 g | 150 g | (Melbourne-Thomas et al., 2011a) |
| hdam_C_ | The factor by which coral cover is reduced by each hurricane event | 0.002 – 0.7 | 0.49 | (Dollar and Tribble, 1993) |
| hdam_M_ | The factor by which macroalgal cover is reduced by each hurricane event | 0.002 – 0.7 | 0.49 | (Dollar and Tribble, 1993) |
| cm_C_ | The factor by which coral cover is reduced by each coral mortality event | 0.3-0.6 | 0.3 | Assumption based on dominant coral genera (Marshall and Baird, 2000) |
| dfdam_C_ | The factor by which coral cover is reduced annually due to destructive fishing activity | 0.004 – 0.032 | 0.004 – 0.032 | (Melbourne-Thomas et al., 2011a) |
| rnut_M_ | The scaling factor for the growth rate of macroalgae under nutrification | 2 – 7 | 2 – 7 | (Melbourne-Thomas et al., 2011a) |
| lnut_C_ | The scaling factor for coral larval production under nutrification | 0 – 0.25 | 0 – 0.25 | (Melbourne-Thomas et al., 2011a) |
| rsed_C_ | The scaling factor for coral growth rate under sedimentation | 0.2 | 0.2 | (Melbourne-Thomas et al., 2011a) |
| dsed_C_ | The increase in coral mortality rate (per year) under sedimentation | 0 – 0.2 | 0 – 0.2 | (Melbourne-Thomas et al., 2011a) |
| drecsed_C_ | The scaling factor for the survival of coral recruits under sedimentation | 0.6 | 0.6 | (Melbourne-Thomas et al., 2011a) |
| ɛsed_C_ | The recruitment rate of coral onto macroturf under sedimentation | 0 | 0 | (Melbourne-Thomas et al., 2011a) |
| Frec | Scaling factor of fish recruitment survival: *Frec* = *aC* / [1 + (*a/b) * C^d^*], where *C*  is coral cover, and *a, b* and *d*are fitted parameters. |  | *a* = 101  *b* = 1.1  *d* = 0.83 | (Gurney et al., 2013) |
| Fpred | Scaling factor of herbivore availability to predation: refuge= min(H,F_pred_*C) ], where *C*  is coral cover and *H* herbivore biomass |  | 12,500 | (Liu and Xing, 2012; Rogers et al., 2014) |

# Supplementary Text S3: Model calibration and validation

Model calibration and validation followed two steps: (1) instantiate the model and run it for 40 years to assess whether modeled output data remained stable for all functional groups (Fig. S3.1); and (2) validate the model by evaluating modeled trajectories against reef dynamics observed over the past 25 years, given a known series of stressors in the Maui Nui area. For the second step, empirical values for reef state variables were assembled at each site in 1985 and a historical timeline of stressors known to have affected the sites during 1987 – 2008 was constructed. The model was forced with these historical stressors and model outcome (Fig. S3.2) was compared with empirical data of reef state (Figs. S3.3).

The first step in model validation shows that all groups stabilized after 25 years with piscivores increasing in the absence of fishing and herbivores decreasing with an increase in piscivores. The model stayed stable for the remaining duration of the model simulation (Fig. S3.1).


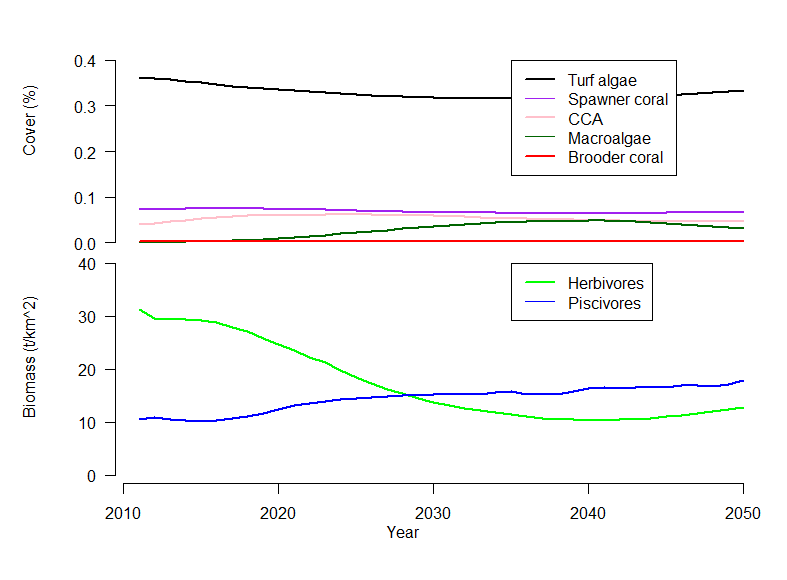


**Figure S3.1.** Trajectories of (*top panel*) benthic and (*bottom panel*) fish groups of a 40-year simulation without any stressors to the coral reef components. CCA is Crustose Coralline Algae

**Historical timeline of stressors at Maui Nui during 1985 – 2010**

Historical stressors, which vary between sites, include increased ocean temperatures leading to coral bleaching events, strong wave events, sedimentation, nutrification, and fishing.

***Bleaching-related coral mortalities***

No severe bleaching events were recorded prior to 2015 in Maui Nui. A very mild bleaching event occurred in the summer of 1996, in which < 10% of the corals bleached and all recovered within months. Hence, no coral mortality events due to bleaching were simulated in historical runs.

***Hurricanes***

Hurricanes are relatively rare in the Hawaiian Islands (Friedlander et al., 2008). Hurricanes come mostly from the southeast (NOAA Pacific Hurricane Center), leaving Maui Nui somewhat protected by the large volcanoes on Hawaiʻi Island. However, the reefs around Hawaiʻi endure strong winter wave events from October to April, as well as occasional tsunami events (NOAA Weather Station). Large winter swells originate from the northeast, whereas tsunami waves mostly come from the southeast, albeit much more infrequently. Large wave events were simulated using a randomly selected probability parameter chosen by the model within the constrains given in the base files.

***Sedimentation***

Land-based sediments reach the coastal waters through point-sources (e.g., rivers) and nonpoint sources (e.g., groundwater discharge) (Street et al., 2008). Since the start of the last century, Maui Nui supported extensive sugarcane and pineapple agriculture until 2009. The spatial input of sediments currently in the Maui-Nui area was based on Wedding and Lecky et al.(Wedding et al., 2017). Available land use data for six watersheds in northwest Maui was used to calculate the ratio of the 2010 to 1920 agricultural land (Stock et al., 2016). This ratio (3.60) was assumed to be valid for the entire Maui Nui area and current sediment levels were multiplied with the agricultural land ratio.

***Nutrification***

Nutrient levels, like sediments, are linked to land use. Additionally, they are related to human population size. The amount of fertilizer used in agriculture far exceeds nutrient pollution through waste disposal or other sources of nitrification. The spatial input of nutrients currently in the Maui-Nui area was based on Wedding and Lecky et al (Wedding et al., 2017). The 1920 to 2010 ratio for nutrient inputs based on agricultural use was 7.85. However, in the last 30 years, the population of Maui has more than doubled; increases of 30% and 16% were observed in Lānaʻi and Molokaʻi, respectively. The ratio was adjusted in 5-year bins to account for this increase for Maui but since the population of both Lānaʻi and Molokaʻi is less than 5% of the population of Maui Nui not for those islands. The contribution of the human pollution was assumed to be smaller than agricultural land use and the ratio was multiplied by 0.5 (0.5*population increase since 1985). For the 2005 – 2010 bin, agriculture was greatly diminished, and (0.5*agricultural land) ratio was used. For the 2010 – 2015 bin, the value estimated by Falinski et al. (Stock et al., 2016) was used (current condition). These adjustments resulted in the following ratios: 1985 – 1989: 3.93; 1990 – 1994: 5.71; 1995 – 1999: 5.05; 2000 – 2004: 4.82; 2005 – 2009: 3.93; 2010 – 2015: 1.

***Fishing***

In Hawaiʻi, there are two types of fishing pressure: commercial fishing, which is conducted by fishermen who sell a portion (or all) of their catch, and recreational fishing, undertaken for leisure, educational, or subsistence purposes. Recreational fishing landings can greatly exceed the commercial catches (Friedlander and Parrish, 1997; McCoy et al., 2018). Reef fish landings were estimated based on commercial data (DAR, downloaded from <http://www.pifsc.noaa.gov/wpacfin/hi/dar/Pages/hi_data_3.php>) and creel survey data (McCoy et al., 2018). These data sources indicate that reef fish catches have decreased over the 1985 – 2000 time period and subsequently stabilized at around 240 tons per year. Piscivores comprised the bulk of all landings.

**Comparing simulated historical trajectories and known reef state**

Historical stressors reduced piscivorous fish biomass, resulting in a slight increase in herbivorous fish biomass (Fig S3.2). Based on anecdotal evidence, a reduction in piscivorous fish seems accurate; however, the trend of herbivorous fish is not validated due to a lack of time series data. Algal cover increased in the late 1980s to mid 1990s which corresponds with the increase in invasive algae especially (Smith et al., 2002).


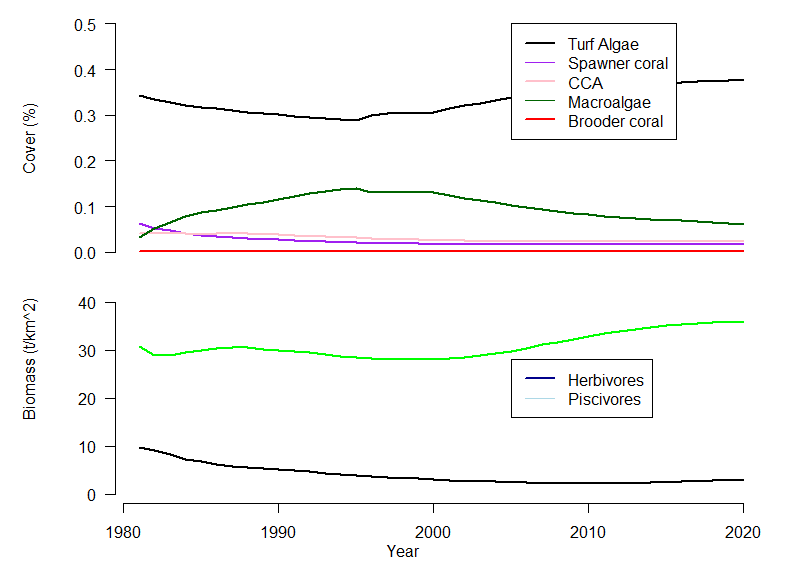


**Figure S3.2.** Trajectories of (*top panel*) benthic cover composition and (*bottom panel*) fish biomass of a 1980 – 2020 hindcast simulation including historic stressors.

Comparing observed and predicted data shows a good correspondence for coral cover with observed data from 3 long-term (1989 ***–*** 2012) sites in Maui (data from Eric Brown, National Park Service, and CRAMP) which was compared to the relative change of model output (Fig. S3.3). Secondarily, a time series of macroalgal cover from 16 sites in West Maui (DAR data 1998 ***–*** 2016) was used. Since the macroalgae time-series starts at 1998, it was assumed that macroalgal cover was the same in the first 10 years. Because no time-series for the entire Maui Nui complex were available, the relative change was evaluated as an indication of the trend for Maui Nui for validation. Comparing observed and predicted data for macroalgal cover, the trend is comparable although absolute values seem to be overestimated by the model (Fig. S3.3).


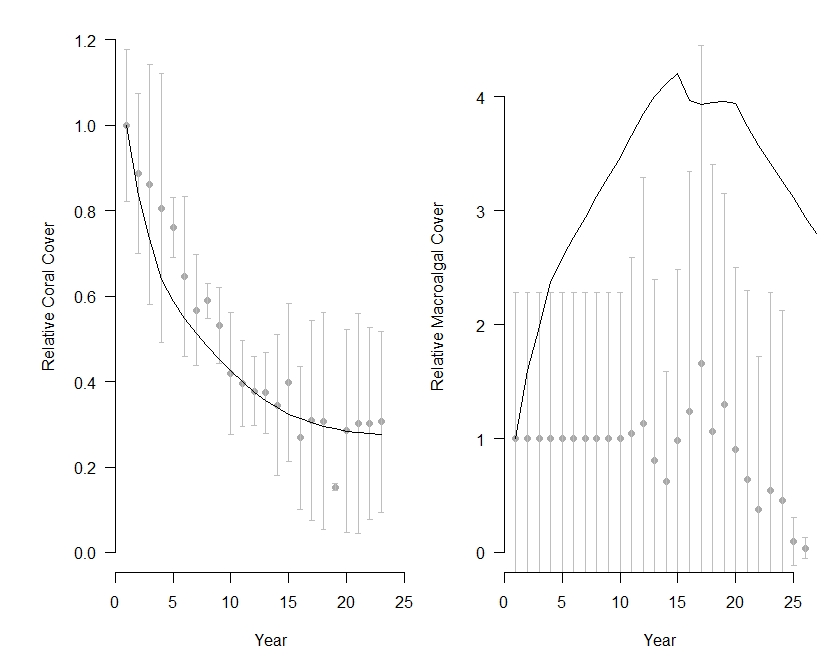


**Figure S3.3.** Observational (grey dots) data and model predicted (solid line) data of the relative change in (*left*) coral cover and (*right*) macroalgal cover in Maui Nui from 1998 – 2015 (with 1998 repeated for the first 10 years due to lack of historic data).

# Supplementary Table S4: Parameters used in Ecological Production Functions (EPF)

| **EPF** | **Parameters** | **Descriptions** |
| --- | --- | --- |
| State of the Reef | Coral cover | Percent cover per pixel; sourced directly from input layers; weighted by a factor of 0.3 |
|  | Macroalgal cover | Percent cover per pixel; sourced directly from input layers; weighted by a factor of 0.15 |
|  | Fish richness | Number of different species in a given pixel; calculated by HIReefSim as a function of fish biomass; weighted by a factor of 0.15 |
|  | Coral richness | Number of different species in a given pixel; calculated by HIReefSim as a function of percent coral cover; weighted by a factor of 0.2 |
|  | Fish abundance | Number of individuals per pixel, calculated by HIReefSim as a function of fish biomass and richness; weighted by a factor of 0.2 |
| Trophic Integrity of the Reef | Ratio of calcifiers to fleshy macroalgae | Ratio of sum of corals and CCA cover divided by the sum of turf and macroalgae cover; weighting factor of 0.5 |
|  |  | Herbivore composition multiplied by trophic level of 2 plus the piscivore composition multiplied by trophic level 4; weighting factor of 0.5 |
| Fisheries Production | Resource fish biomass | Total biomass (kg/km^2^) of key harvested fish species as a fraction of total fish biomass |
| Fisheries Landings | Landed resource fish biomass | Total biomass (kg/km^2^) of annually landed fishes |

# Supplementary Figures S5: Forecast trajectories under different management and climate-related stressors


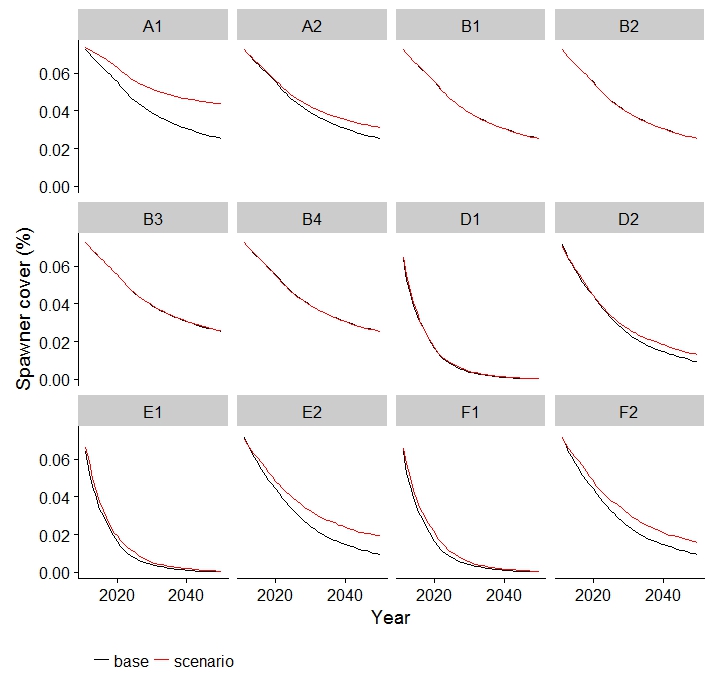


**Figure S5.1.** Spawner cover trajectories under different scenario simulations. Base is “Current Management” for scenarios A and B and current management under severe climate-related stressors for scenarios D1, E1, and F1 and less severe climate-related stressors for scenarios D2, E2, and F2.


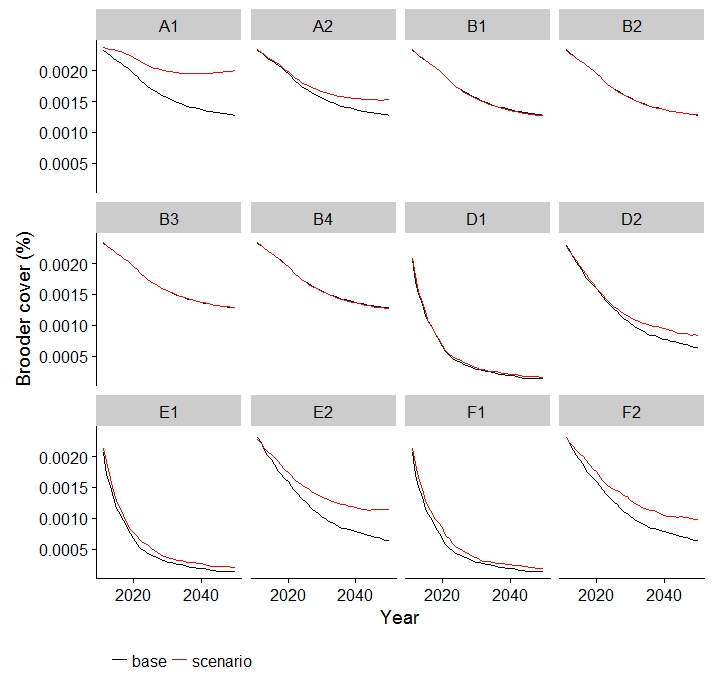


**Figure S5.2.** Brooder cover trajectories under different scenario simulations. Base is “Current Management” for scenarios A and B and current management under severe climate-related stressors for scenarios D1, E1, and F1 and less severe climate-related stressors for scenarios D2, E2, and F2.


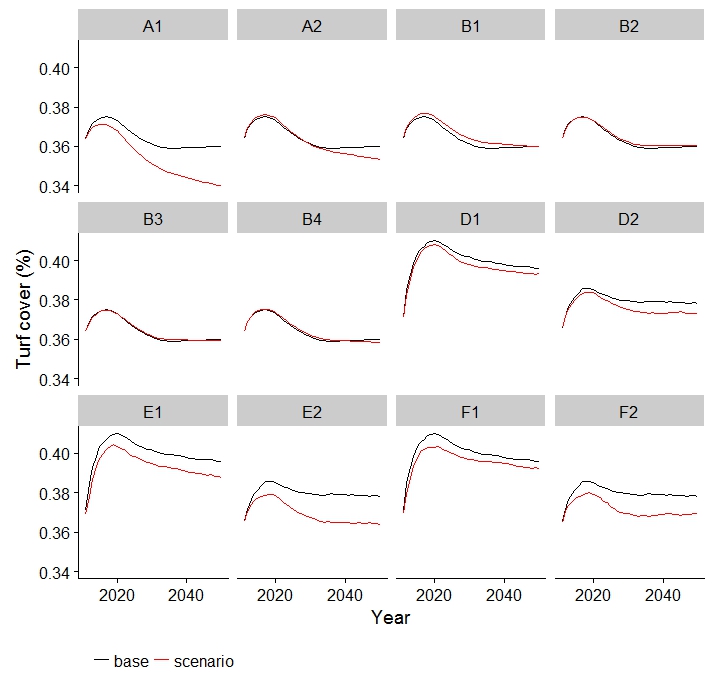


**Figure S5.3.** Turf cover trajectories under different scenario simulations. Base is “Current Management” for scenarios A and B and current management under severe climate-related stressors for scenarios D1, E1, and F1 and less severe climate-related stressors for scenarios D2, E2, and F2.


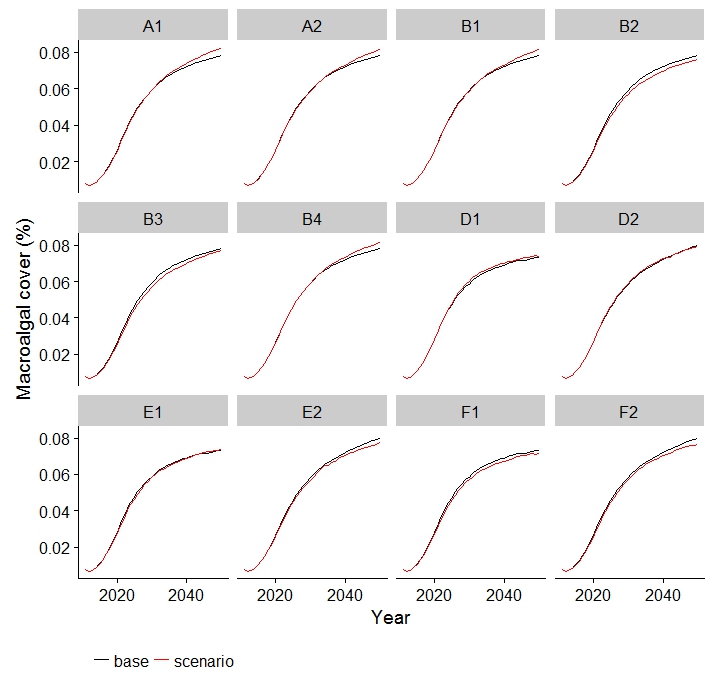


**Figure S5.4.** Macroalgal cover trajectories under different scenario simulations. . Base is “Current Management” for scenarios A and B and current management under severe climate-related stressors for scenarios D1, E1, and F1 and less severe climate-related stressors for scenarios D2, E2, and F2.


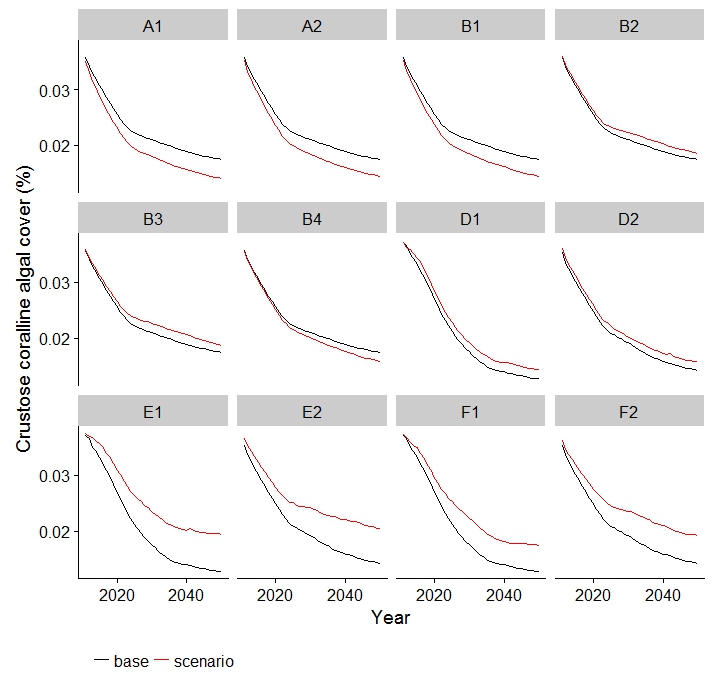


**Figure S5.5.** Crustose coralline algal cover trajectories under different scenario simulations. Base is “Current Management” for scenarios A and B and current management under severe climate-related stressors for scenarios D1, E1, F1 and less severe climate-related stressors for scenarios D2, E2, F2.


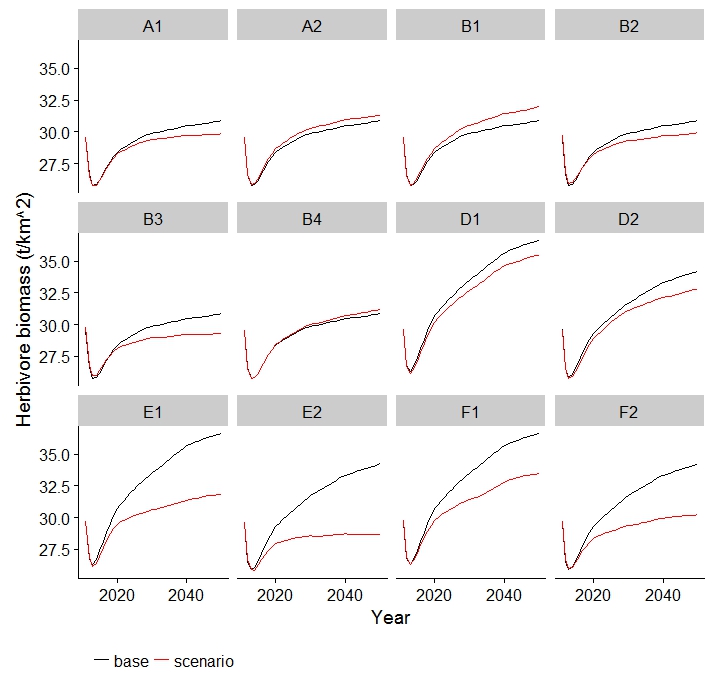


**Figure S5.6.** Herbivorous fishes biomass trajectories under different scenario simulations. . Base is “Current Management” for scenarios A and B and current management under severe climate-related stressors for scenarios D1, E1, F1 and less severe climate-related stressors for scenarios D2, E2, F2.


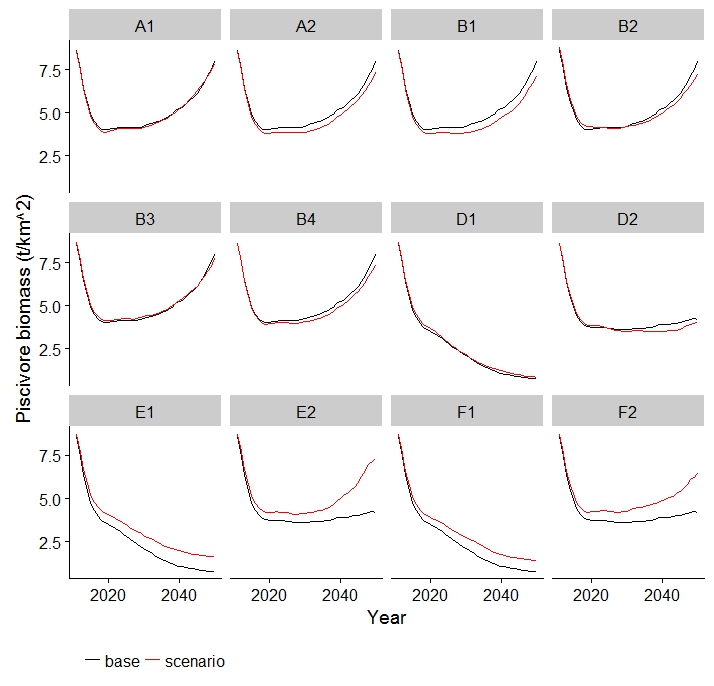


**Figure S5.7.** Piscivorous fishes biomass trajectories under different scenario simulations. . Base is “Current Management” for scenarios A and B and current management under severe climate-related stressors for scenarios D1, E1, F1 and less severe climate-related stressors for scenarios D2, E2, F2.

# Supplementary Figures S6: Spatially explicit projected relative change in three ecosystem services from 2010 *–* 2040

**State of the Reef (SOR)**


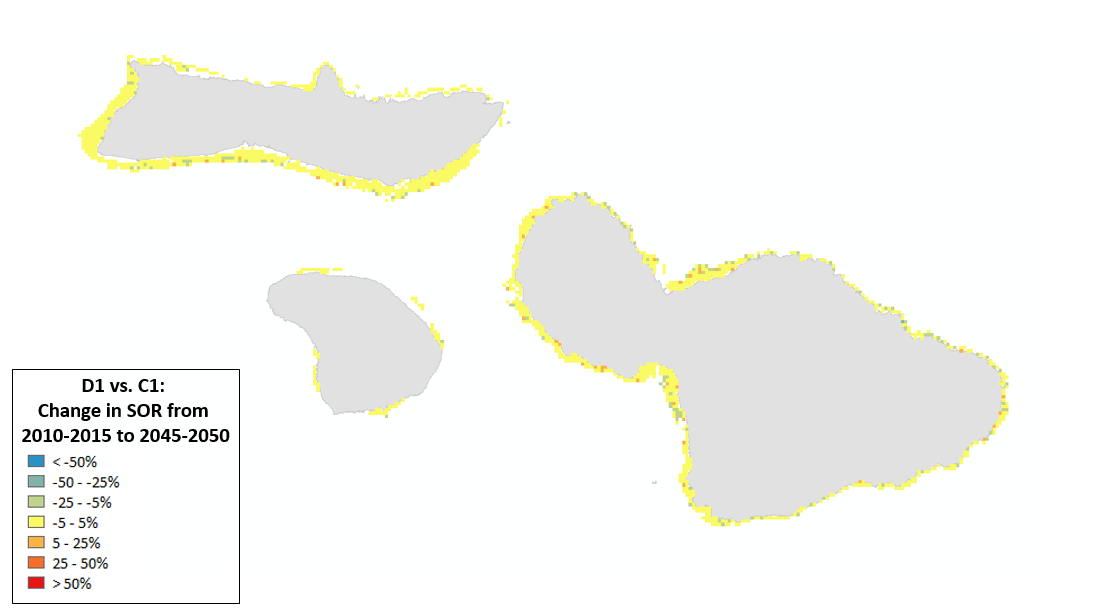


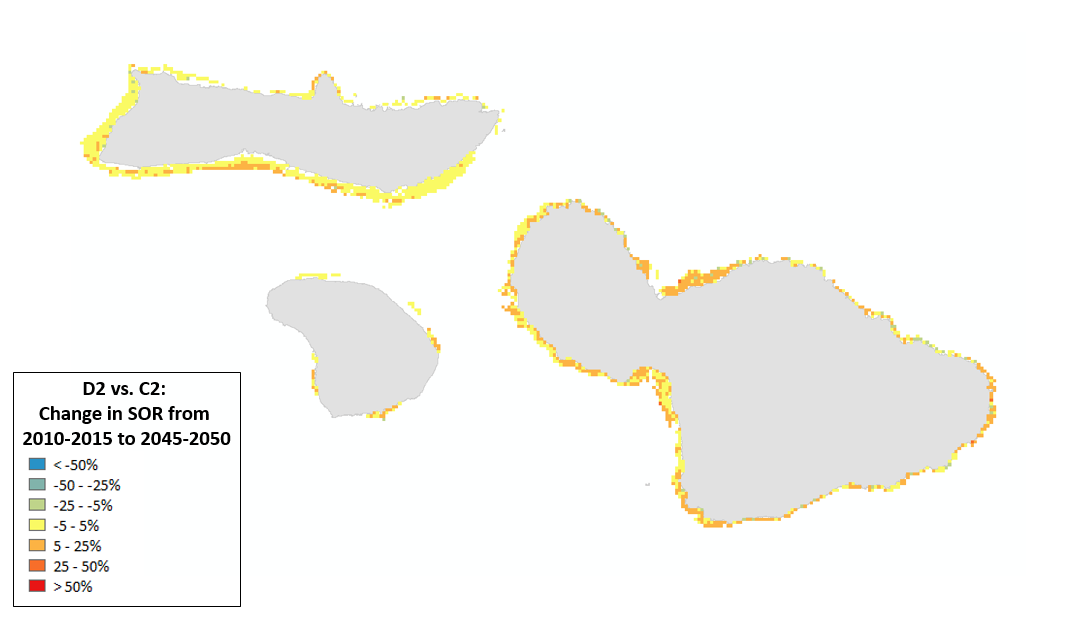

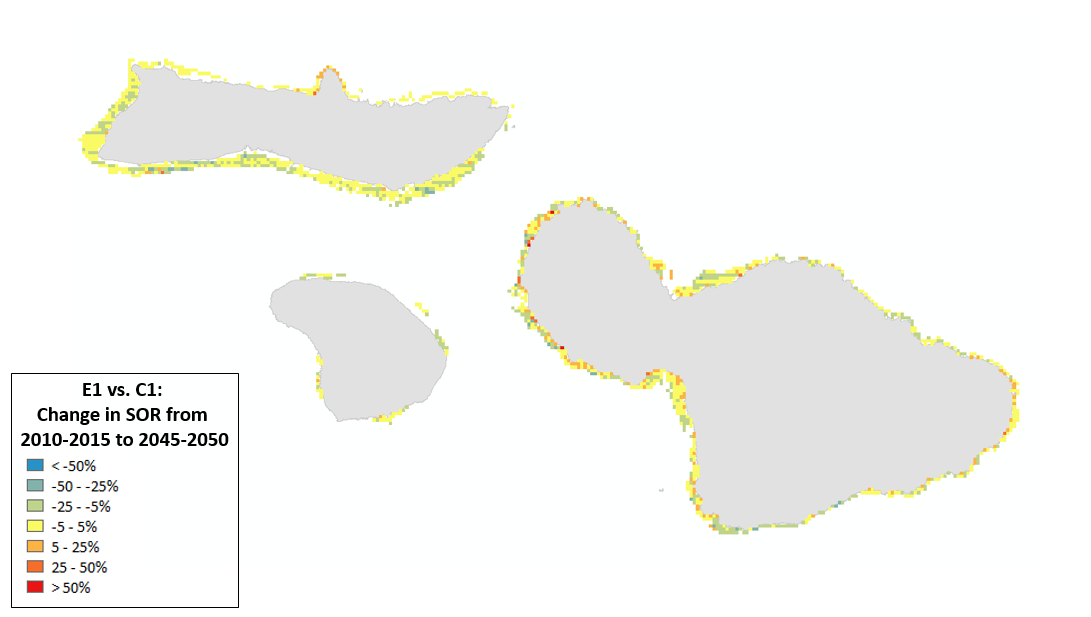

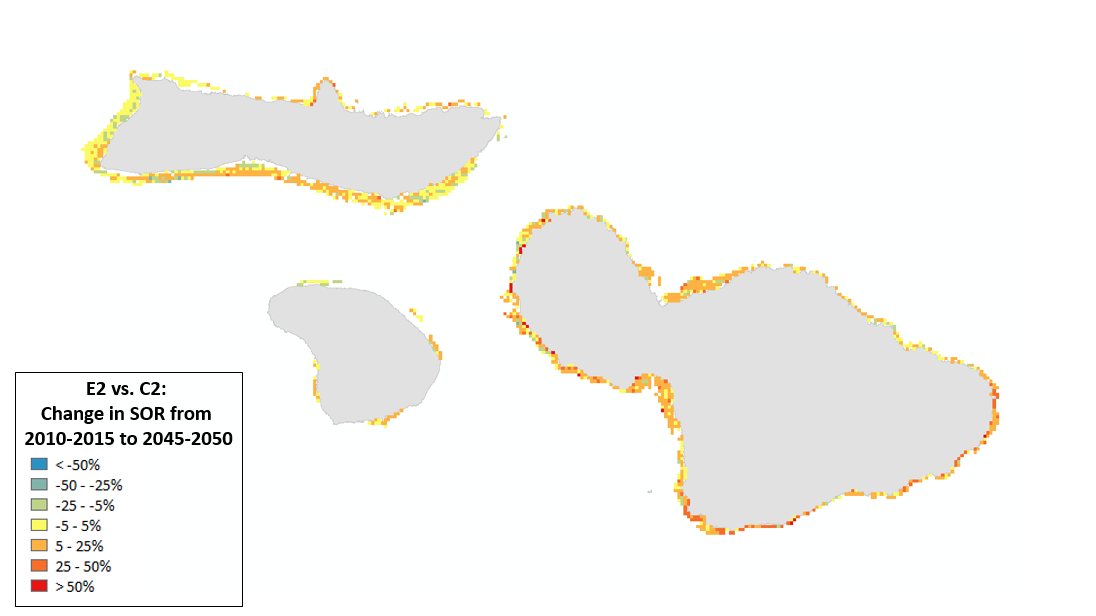

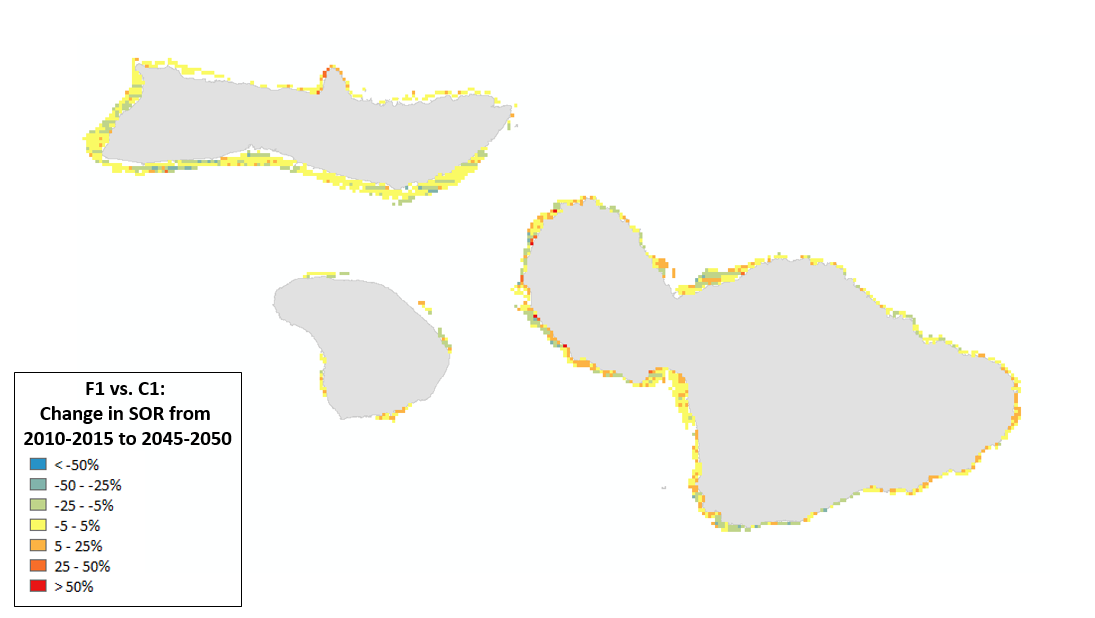

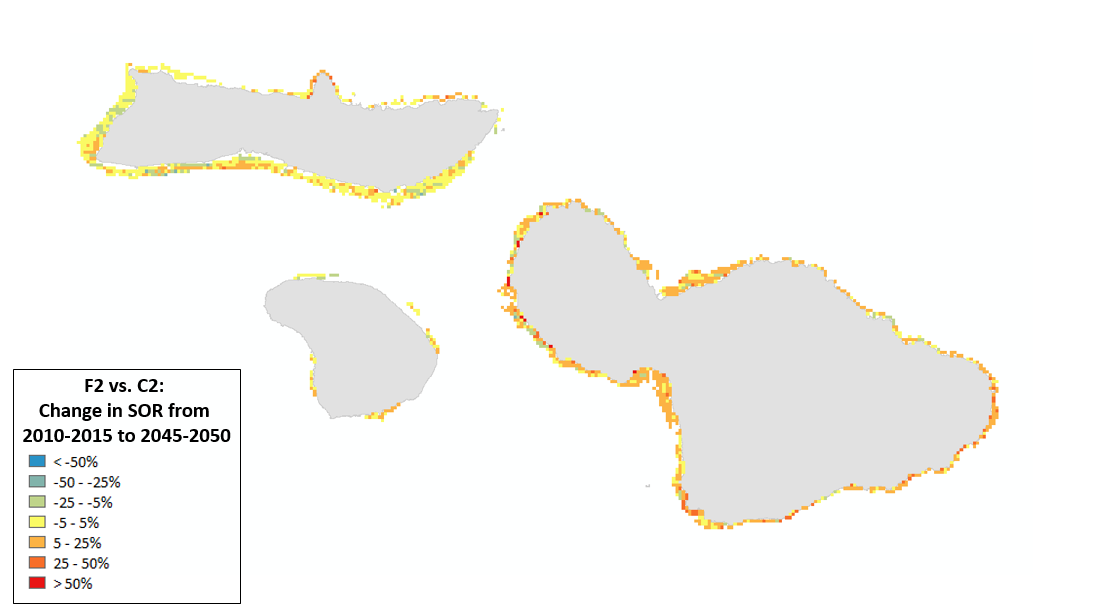


**Trophic Integrity of the Reef (TI)
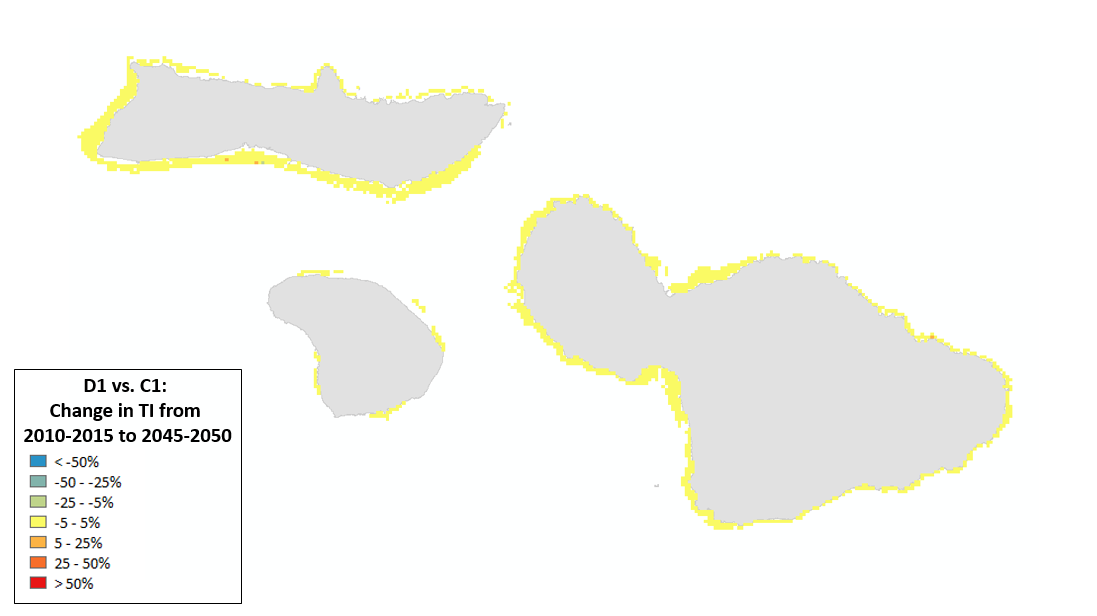

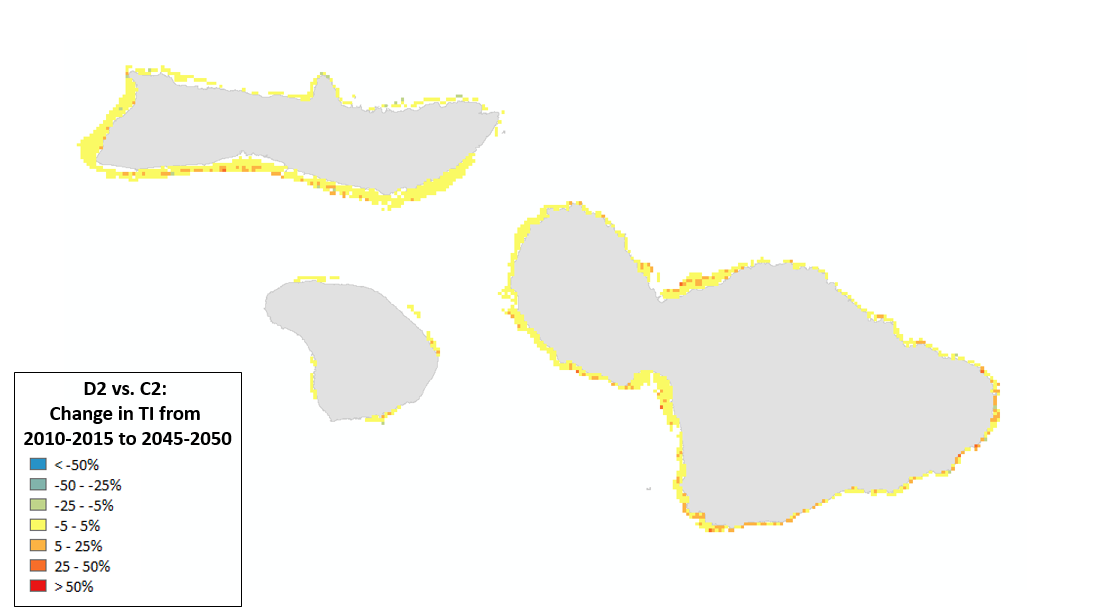
**


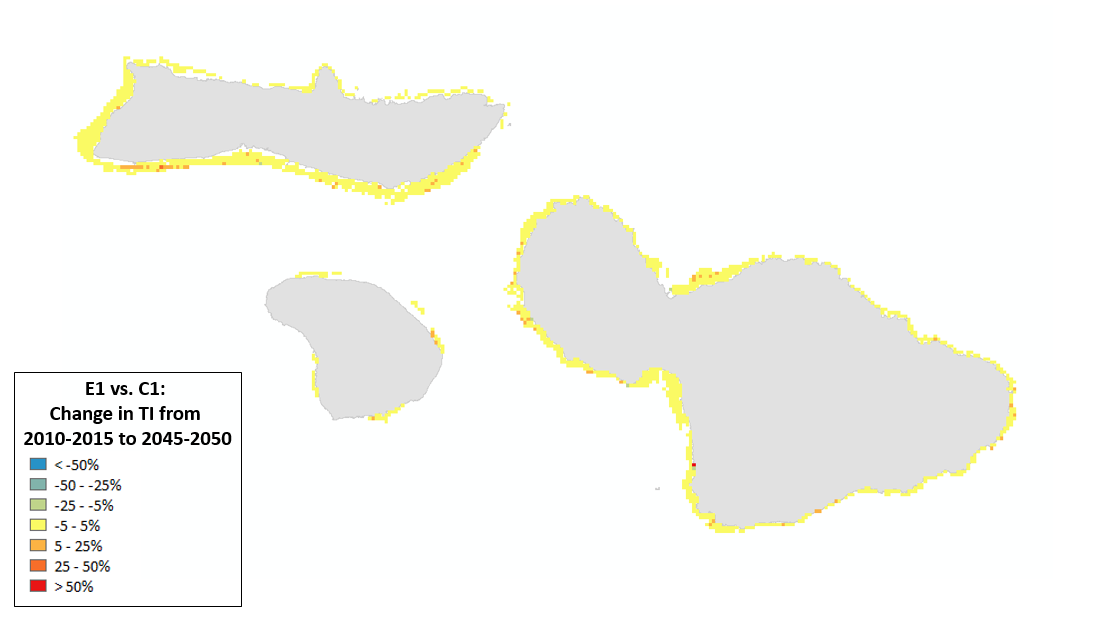

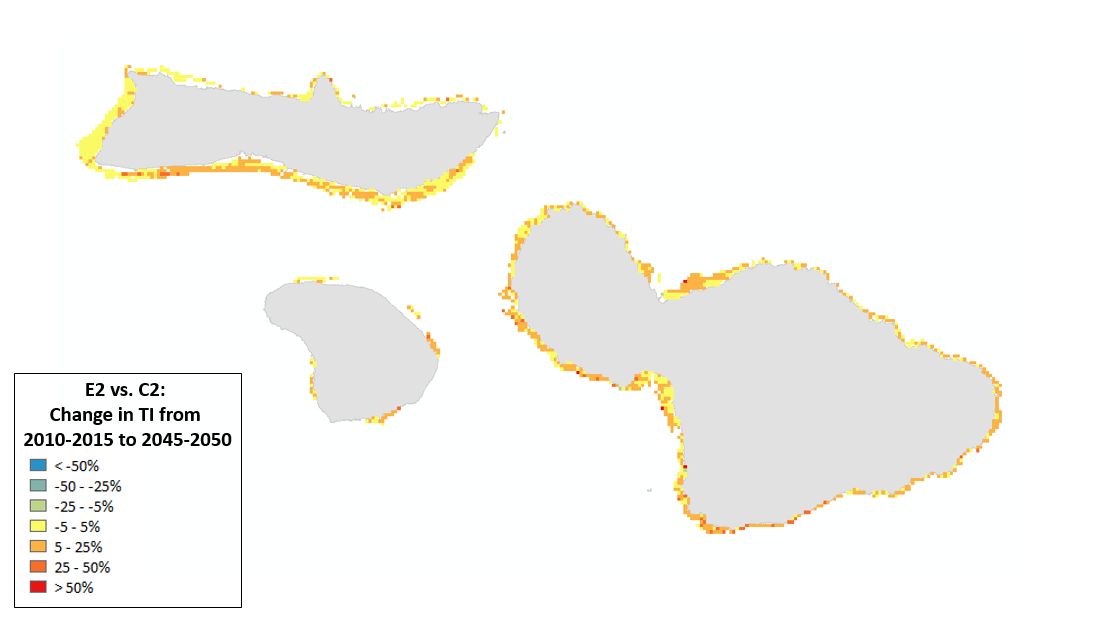


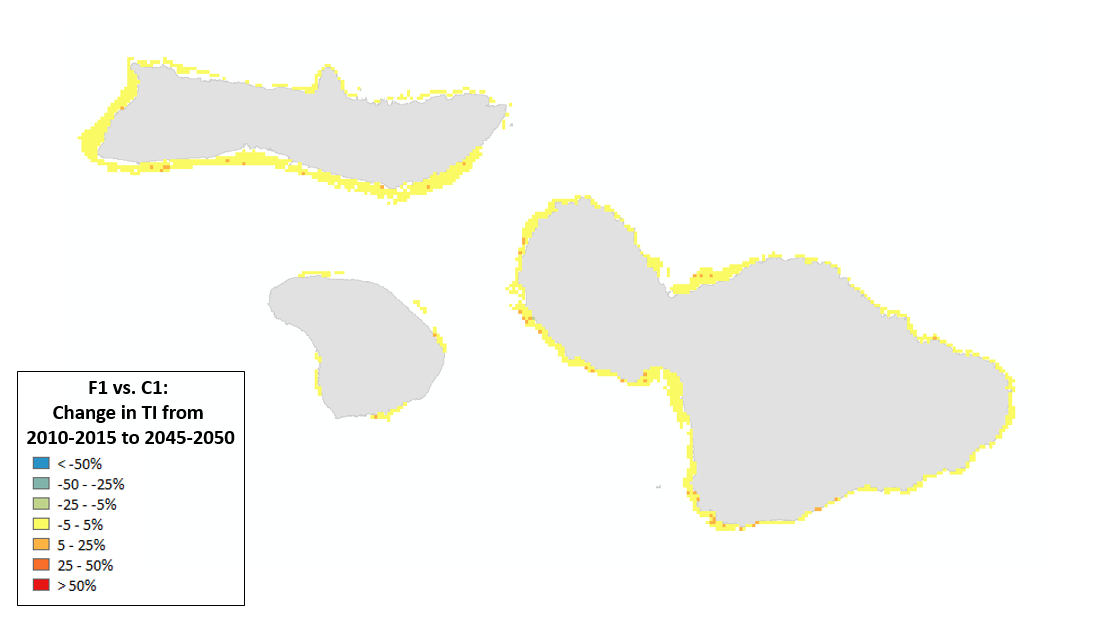

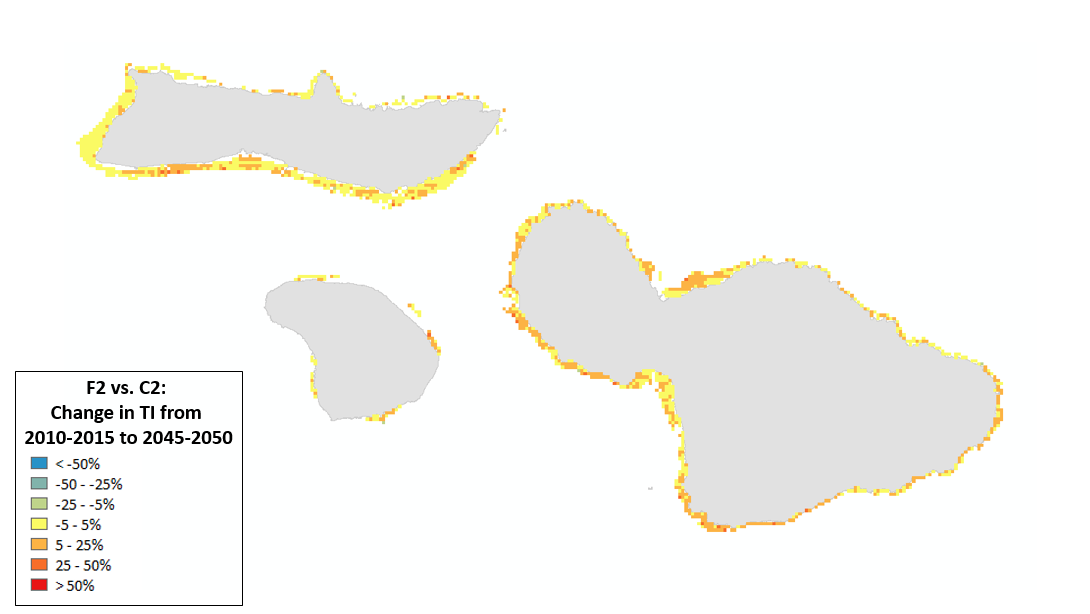


**Fisheries Production (FP)**

**
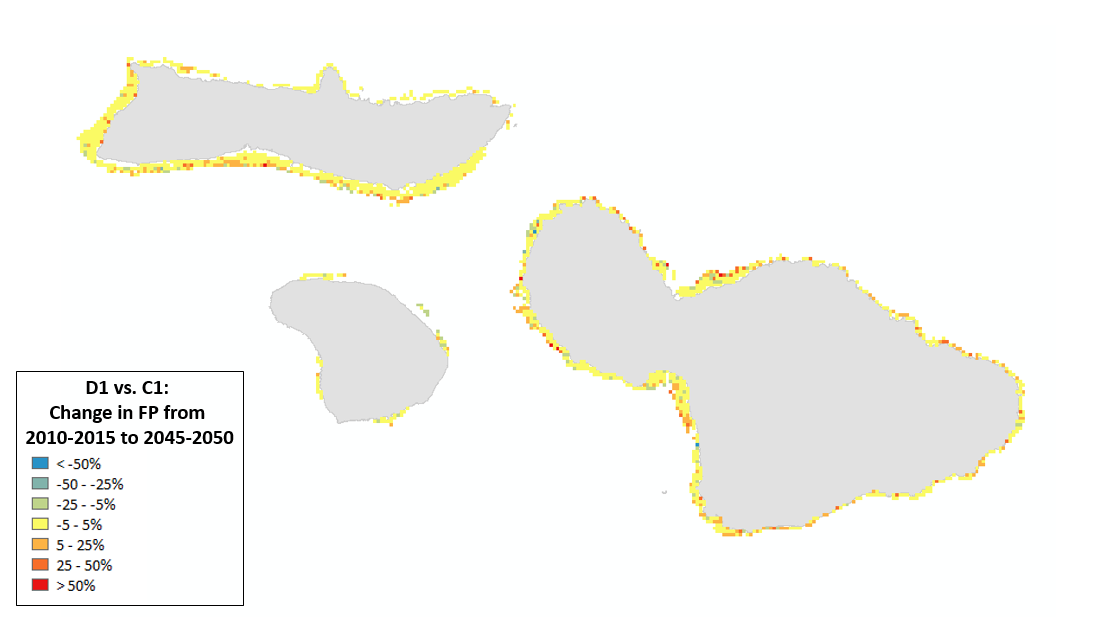
**

**
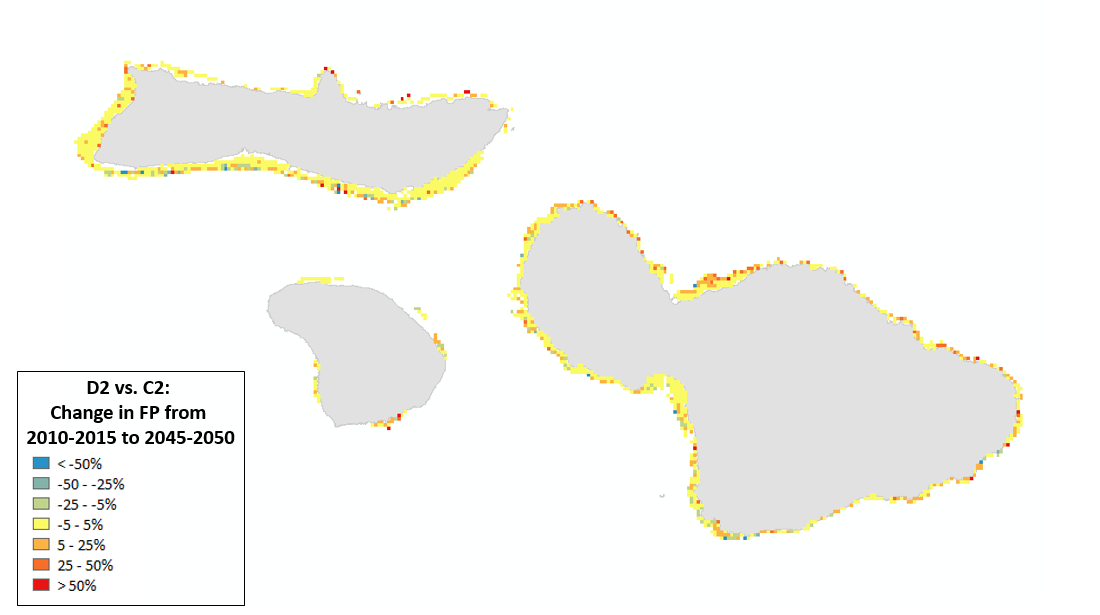
**

**
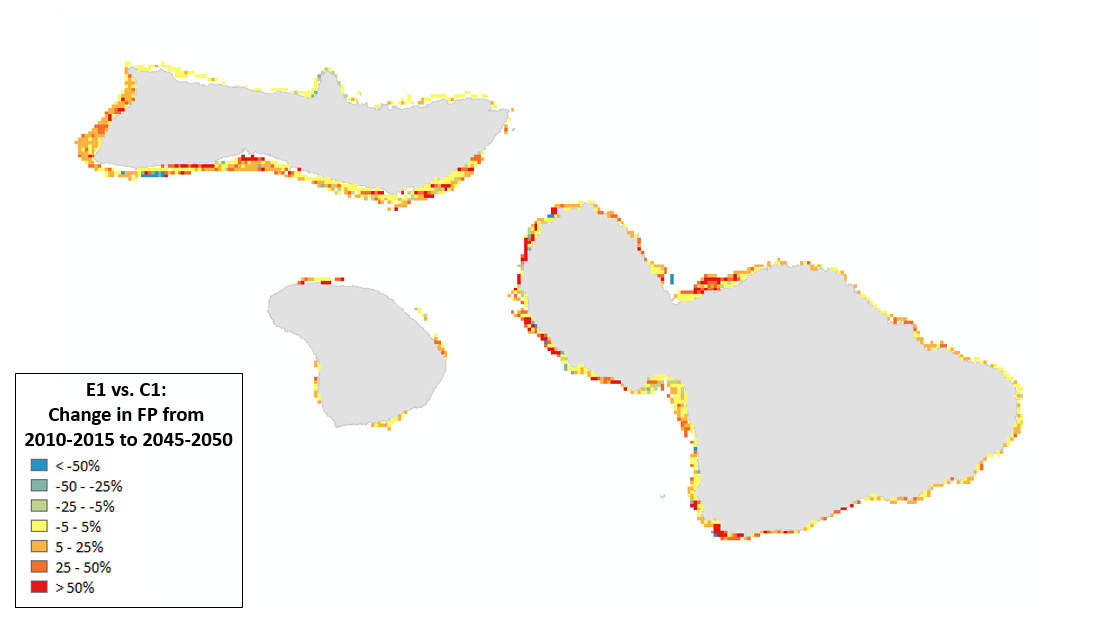
**

**
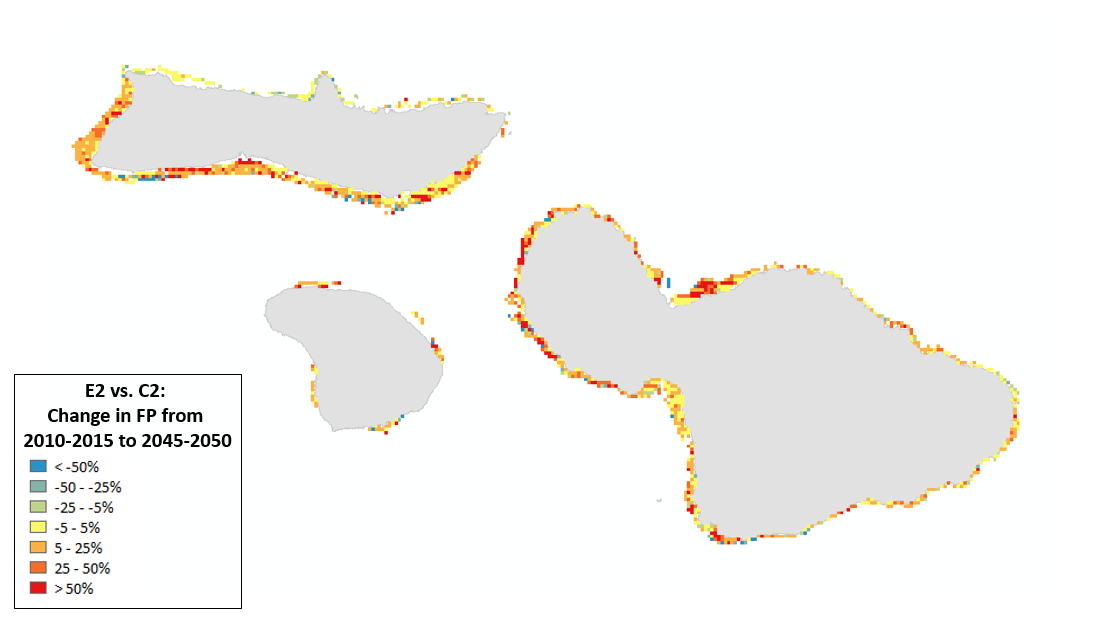

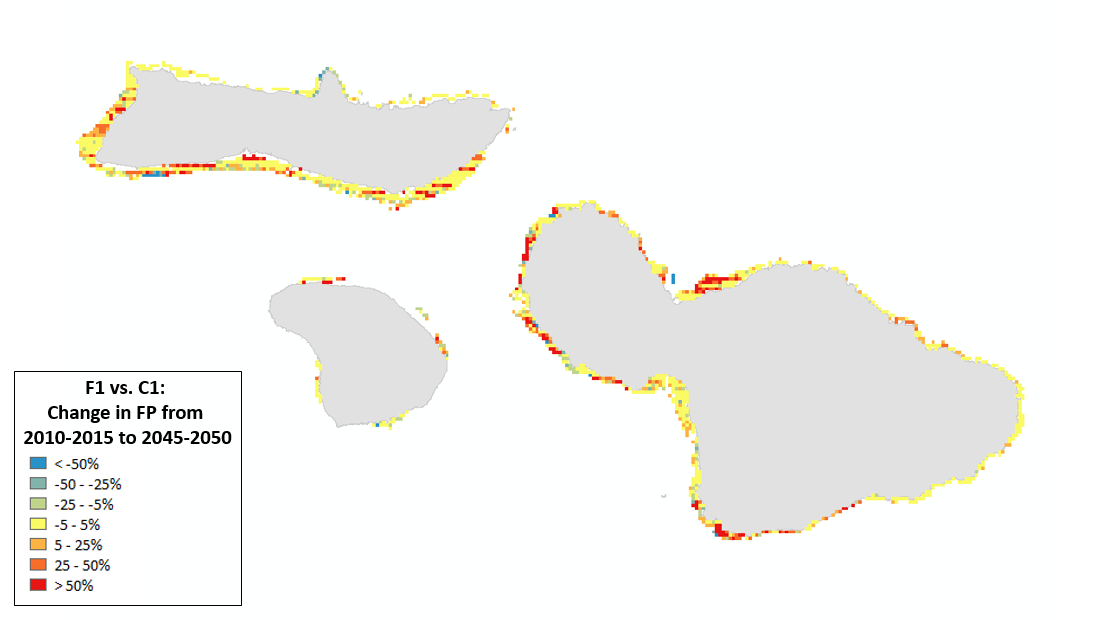
**

**
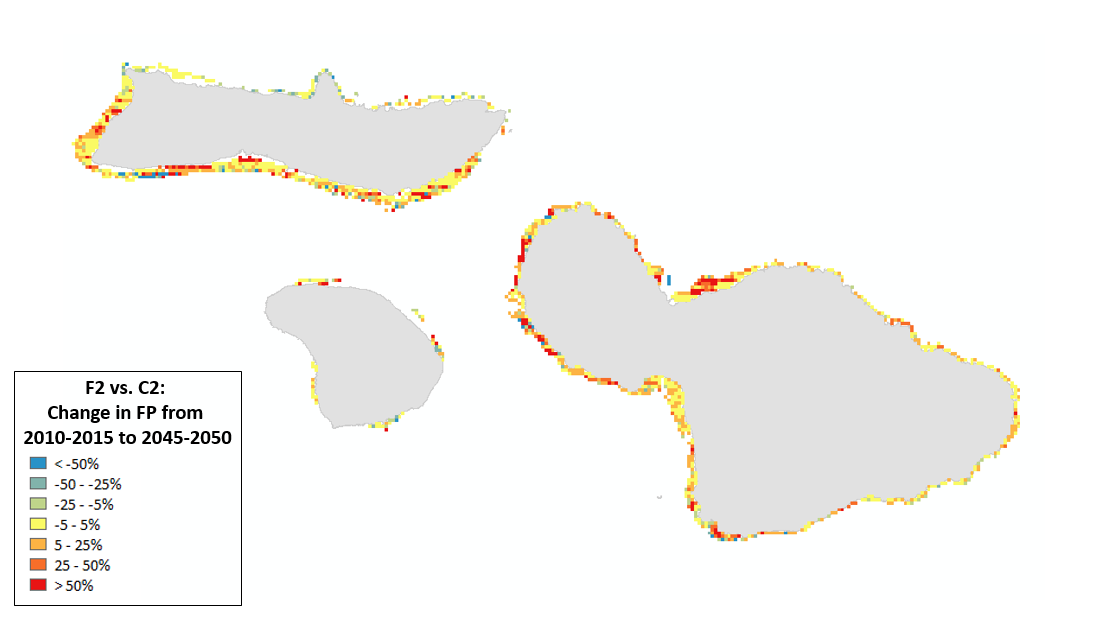
**

# Supplementary Text S7: References in all appendices

Birkeland, C. (1977). The importance of rate of biomass accumulation in early successional stages of benthic communities to the survival of coral recruits. in *Proceedings of the 3rd International Coral Reef Symposium*, ed. D. L. Taylor (Miami, FL: Proc 3rd Intl Coral Reef Symp), 15–21.

Birrell, C. L., McCook, L. J., and Willis, B. L. (2005). Effects of algal turfs and sediment on coral settlement. *Mar. Pollut. Bull.* 51, 408–414.

Box, S. J., and Mumby, P. J. (2007). Effect of macroalgal competition on growth and survival of juvenile Caribbean corals. *Mar. Ecol. Prog. Ser.* 342, 139–149.

Dollar, S. J., and Tribble, G. W. (1993). Recurrent storm disturbance and recovery: a long-term study of coral communities in Hawaii. *Coral Reefs* 12, 223–233. doi:10.1007/BF00334481.

Forsman, Z. H., Concepcion, G. T., Haverkort, R. D., Shaw, R. W., Maragos, J. E., and Toonen, R. J. (2010). Ecomorph or endangered coral? DNA and microstructure reveal hawaiian species complexes: Montipora dilatata/ flabellata/turgescens & M. patula/verrilli. *PLoS One* 5, e15021. doi:10.1371/journal.pone.0015021.

Friedlander, A. M., Aeby, G., Brainard, R. E., Brown, E. K., Chaston, K., Clark, A., et al. (2008). The state of coral reef ecosystems of the Main Hawaiian Islands. *State Coral Reef Ecosyst. United States Pacific Free. Assoc. States 2008*, 219–253.

Friedlander, A. M., and Parrish, J. D. (1997). Fisheries harvest and standing stock in a Hawaiian Bay. *Fish. Res.* 32, 33–50.

Fung, T. C. (2009). Local scale models of coral reef ecosystems for scenario testing and decision support. *Fac. Maths Phys. Sci.* PhD. Available at: https://reefscenarios.org/Resources.

Gurney, G. G., Melbourne-Thomas, J., Geronimo, R. C., Aliño, P. M., and Johnson, C. R. (2013). Modelling coral reef futures to inform management: can reducing local-scale stressors conserve reefs under climate change? *PLoS One* 8. doi:10.1371/journal.pone.0080137.

Klumpp, D. W., and McKinnon, A. D. (1992). Community structure, biomass and productivity of epilithic algal communities on the Great Barrier Reef: Dynamics at different spatial scales. *Mar. Ecol. Prog. Ser. Oldend.* 86, 77–89.

Kolinski, S. P. P. (2007). Recovery Projections for Scleractinian Corals Injured in the M/V Cape Flattery Incident, Oahu, Hawaii, 2005 . Honolulu: NOAA Fisheries, Pacific Islands Regional Office Available at: https://casedocuments.darrp.noaa.gov/southwest/capeflattery/pdf/RecoveryProjections.pdf.

Koop, K., Booth, D., Broadbent, A., Brodie, J., Bucher, D., Capone, D., et al. (2001). ENCORE: The effect of nutrient enrichment on coral reefs. Synthesis of results and conclusions. *Mar. Pollut. Bull.* 42, 91–120. doi:10.1016/S0025-326X(00)00181-8.

Lirman, D. (2001). Competition between macroalgae and corals: effects of herbivore exclusion and increased algal biomass on coral survivorship and growth. *Coral Reefs* 19, 392–399.

Liu, X., and Xing, Y. (2012). Qualitative analysis for a predator prey system with holling type III functional response and prey refuge. *Discret. Dyn. Nat. Soc.* 2012. doi:10.1155/2012/678957.

Marshall, P. A., and Baird, A. H. (2000). Bleaching of corals on the Great Barrier Reef: differential susceptibilities among taxa. *Coral Reefs* 19, 155–163.

McCook, L. (2001). Competition between corals and algal turfs along a gradient of terrestrial influence in the nearshore central Great Barrier Reef. *Coral Reefs* 19, 419–425.

McCoy, K. S., Williams, I. D., Friedlander, A. M., Hongguang, M., Teneva, L. T., and Kittinger, J. N. (2018). Estimating nearshore coral reef-associated fisheries production from the main Hawaiian Islands using commercial and non-commercial data. *PloS one Press*, 1–13. doi:10.1371/journal.pone.0195840.

Melbourne-Thomas, J., Johnson, C. R., and Fulton, E. A. (2011a). Regional-scale scenario analysis for the Meso-American Reef system: Modelling coral reef futures under multiple stressors. *Ecol. Modell.* 222, 1756–1770.

Melbourne-Thomas, J., Johnson, C. R., Fung, T., Seymour, R. M., Chérubin, L. M., Arias-González, J. E., et al. (2011b). Regional-scale scenario modeling for coral reefs: a decision support tool to inform management of a complex system. *Ecol. Appl.* 21, 1380–1398. doi:10.1890/09-1564.1.

Minton, D., and Ph, D. (2013). Review of Growth Rates for Indo-Pacific Corals Prepared by.

Richmond, R. H., and Hunter, C. L. (1990). Reproduction and recruitment of corals: Comparisons among the Caribbean, the Tropical Pacific, and the Red Sea. *Mar. Ecol. Prog. Ser. Oldend.* 60, 185–203.

Rogers, A., Blanchard, J. L., and Mumby, P. J. (2014). Vulnerability of Coral Reef Fisheries to a Loss of Structural Complexity. *Curr. Biol.* 24, 1000–1005. doi:10.1016/j.cub.2014.03.026.

Smith, J. E. (2006). Algal blooms in North Kiehi, Maui: Assessing the links between land-based nutrients and algal abundance and distribution. Kihei: In: Technical Report to the City and County of Maui, HI.

Smith, J. E., Hunter, C. L., and Smith, C. M. (2002). Distribution and reproductive characteristics of nonindigenous and invasive marine algae in the Hawaiian Islands. *Pac. Sci*. 56, 299-315. doi: 10.1353/psc.2002.0030

Smith, J. E., Smith, C. M, and Hunter, C. L. (2001). An experimental analysis of the effects of herbivory and nutrient enrichment on benthic community dynamics on a Hawaiian reef. *Coral Reefs* 19, 332–342. doi:10.1007/s003380000124.

Sparks, R., Stone, K., and White, D. (2011). Maui and Lanai Monitoring Report. Wailuku, Maui.

Stock, J. D., Falinski, K. A., and Callender, T. (2016). Reconnaissance sediment budget for selected watersheds of West Maui, Hawai‘i. Reston, VA doi:10.3133/ofr20151190.

Storlazzi, C. D., Brown, E. K., and Field, M. E. (2006). The application of acoustic Doppler current profilers to measure the timing and patterns of coral larval dispersal. *Coral Reefs* 25, 369–381.

Street, J. H., Knee, K. L., Grossman, E. E., and Paytan, A. (2008). Submarine groundwater discharge and nutrient addition to the coastal zone and coral reefs of leeward Hawai’i. *Mar. Chem.* 109, 355–376.

Veron, J. E. N., and Stafford-Smith, M. G. (2002). Coral ID.

Wedding, L. M. L. M., Lecky, J. H., Gove, J., Walecka, H. R. H. R., Donovan, M. K. M. K., Williams, G. J. G. J. G. J., et al. (2017). Advancing the Integration of Spatial Data to Map Human and Natural Drivers on Coral Reefs. *PLoS One*, 1–29. doi:10.1371/journal.pone.0189792.

Weijerman, M., Fulton, E. A., and Parrish, F. A. (2013). Comparison of coral reef ecosystems along a fishing pressure gradient. *PLoS One* 8, e63797. doi:10.1371/journal.pone.0063797.

Williams, I. D., Baum, J. K., Heenan, A., Hanson, K. M., Nadon, M. O., and Brainard, R. E. (2015). Human, oceanographic and habitat drivers of central and western Pacific coral reef fish assemblages. *PLoS One* 10, e0120516.

Wren, J. L. K., and Kobayashi, D. R. (2016). Exploration of the “larval pool”: development and ground-truthing of a larval transport model off leeward Hawai‘i. *PeerJ* 4, e1636. doi:10.7717/peerj.1636.
